# Genetic architecture of root and shoot ionomes in rice (*Oryza sativa* L.)

**DOI:** 10.1007/s00122-021-03848-5

**Published:** 2021-05-20

**Authors:** Joshua N. Cobb, Chen Chen, Yuxin Shi, Lyza G. Maron, Danni Liu, Mike Rutzke, Anthony Greenberg, Eric Craft, Jon Shaff, Edyth Paul, Kazi Akther, Shaokui Wang, Leon V. Kochian, Dabao Zhang, Min Zhang, Susan R. McCouch

**Affiliations:** 1 Plant Breeding and Genetics Section, School of Integrative Plant Science, Cornell University, Ithaca, NY 14853-1901 USA; 2 Department of Statistics, Purdue University, West Lafayette, IN 47907-2054 USA; 3 Soil and Crop Sciences Section, School of Integrative Plant Science, Cornell University, Ithaca, NY 14853-1901 USA; 4 Robert W. Holley Center for Agriculture and Health, United States Department of Agriculture-Agricultural Research Service, Ithaca, NY 14853-1901 USA; 5 GeneFlow, Inc, Centreville, VA 20120 USA; 6 Present Address: RiceTec Inc, Alvin, TX 77511 USA; 7 Present Address: Ausy Consulting, Esperantolaan 8, 3001 Heverlee, Belgium; 8 Present Address: Bayesic Research, LLC, 452 Sheffield Rd, Ithaca, NY 14850 USA; 9 Present Address: Department of Plant Breeding, South China Agriculture University, Guangdong, 510642 China; 10 Present Address: Global Institute for Food Security, University of Saskatchewan, Saskatoon, SK S7N 4J8 Canada

**Keywords:** Genome-wide association (GWA), QTL, Multi-marker analysis, Ion, Micronutrient, Haplotype, Candidate gene

## Abstract

**Key message:**

**Association analysis for ionomic concentrations of 20 elements identified independent genetic factors underlying the root and shoot ionomes of rice, providing a platform for selecting and dissecting causal genetic variants.**

**Abstract:**

Understanding the genetic basis of mineral nutrient acquisition is key to fully describing how terrestrial organisms interact with the non-living environment. Rice (*Oryza sativa* L.) serves both as a model organism for genetic studies and as an important component of the global food system. Studies in rice ionomics have primarily focused on above ground tissues evaluated from field-grown plants. Here, we describe a comprehensive study of the genetic basis of the rice ionome in both roots and shoots of 6-week-old rice plants for 20 elements using a controlled hydroponics growth system. Building on the wealth of publicly available rice genomic resources, including a panel of 373 diverse rice lines, 4.8 M genome-wide single-nucleotide polymorphisms, single- and multi-marker analysis pipelines, an extensive tome of 321 candidate genes and legacy QTLs from across 15 years of rice genetics literature, we used genome-wide association analysis and biparental QTL analysis to identify 114 genomic regions associated with ionomic variation. The genetic basis for root and shoot ionomes was highly distinct; 78 loci were associated with roots and 36 loci with shoots, with no overlapping genomic regions for the same element across tissues. We further describe the distribution of phenotypic variation across haplotypes and identify candidate genes within highly significant regions associated with sulfur, manganese, cadmium, and molybdenum. Our analysis provides critical insight into the genetic basis of natural phenotypic variation for both root and shoot ionomes in rice and provides a comprehensive resource for dissecting and testing causal genetic variants.

**Supplementary Information:**

The online version contains supplementary material available at 10.1007/s00122-021-03848-5.

## Introduction

The acquisition and metabolism of elements (ions) are of seminal importance to all living things. As sessile organisms, plants have a particularly complex challenge; they must explore the soil environment to find and acquire the nutrients that are essential for growth while avoiding toxic or harmful elements, a task made more difficult by the fact that mineral elements are not uniformly distributed over space and time. As such, plants have developed a suite of adaptive and environmentally responsive mechanisms that allow them to interact with the physical, chemical, and biological components of the soil environment to acquire and internally translocate mineral elements to sustain their own growth and development (Williams and Salt 2009).

The ionome is defined as “the mineral nutrient and trace element composition of an organism” and it represents the inorganic component of cellular and organismal systems (Lahner et al. 2003; Salt et al. 2008). Using well-established techniques in analytical chemistry, large plant populations can be sampled with relative ease for multiple elements simultaneously, making it possible to evaluate the plant ionome in different tissues, across developmental stages, and/or in response to different environmental conditions. Screening large, well-genotyped populations allows ionomic data to be used to map genetic loci that are associated with variation in ion concentrations (Atwell et al. 2010; Baxter et al. 2013; Pinson et al. 2015; Yang et al. 2018; Ziegler et al. 2017). Understanding the genetic architecture and underlying functional genomics of the ionome in major crop species provides important insights about the range of phenotypic variation that exists within and between these species and helps scientists explore the complex trade-offs required to optimize productivity in the face of soil and nutrient constraints, climate change, and the need to improve the long-term sustainability of cropping systems.

Several studies focused on the ionome have been performed using mutagenized populations of *Arabidopsis thaliana* (Lahner et al. 2003), *Saccharomyces cerevisiae* (Eide et al. 2005), and the model legume *Lotus japonicas* (Chen et al. 2009a), as well as natural populations of *Arabidopsis thaliana* (Atwell et al. 2010; Buescher et al. 2010; Ghandilyan et al. 2009a,; Ghandilyan et al. 2009b; Rus et al. 2006; Vreugdenhil et al. 2004), *Lotus japonicas* (Chen et al. 2009b; Sanchez et al. 2011), *Mimulus guttatus* (Lowry et al. 2012), *Zea mays* (Asaro et al. 2016; Baxter et al. 2013, 2014), *Sorghum bicolor* (Shakoor et al. 2016), *Triticum aestivum* (Manickavelu et al. 2017), *Gossypium hirsutum* (Pauli et al. 2018), *Glycine max* (Ziegler et al. 2017), potato (Chaparro et al. 2018), a variety of vegetable crops (Watanabe et al. 2016), and *Oryza sativa* (Li et al. 2013; Liu et al. 2020, 2019; Norton et al. 2010a, b, 2012; Pinson et al. 2015; Yang et al. 2018; Zeng et al. 2010; Zhang et al. 2014). In addition, variation in leaf element composition has been broadly evaluated for 44 elements across 138 distinct families of terrestrial plants (Watanabe et al. 2007).

Rice stands out among these, as it is both one of the most important food crops in the world and a powerful model system for investigating the genetic basis of complex trait variation, with implications across a range of cereal species. Rice is an inbreeding species with a small genome and abundant genetic and genomic resources (3000 Rice Genomes Project 2014; Duitama et al. 2015; Huang et al. 2012; International-Rice-Genome-Sequencing-Project 2005; Jackson 1997; Xu et al. 2012; Zhou et al. 2020). The public availability of immortalized rice diversity panels and associated single-nucleotide polymorphism (SNP) datasets (Huang et al. 2012; McCouch et al. 2016) facilitate genotype–phenotype association studies, and a public-access Rice Imputation Server (Wang et al. 2018) enables the integration of genomic data across diverse germplasm and genomic resources for the entire rice research community.

Genetic analysis in rice is framed by the complex demographic history of *O. sativa,* which has resulted in deep population structure (Garris et al. 2005; Huang et al. 2012; McCouch et al. 2016; Molina et al. 2011; Zhou et al. 2020), characterized by two major varietal groups, INDICA and JAPONICA*,* hereafter referred to as clades. The INDICA clade is comprised of the *indica* and *aus* subpopulations*,* and the JAPONICA clade is comprised of the *temperate japonica* and *tropical japonica* subpopulations (Garris et al. 2005; Huang et al. 2012; McCouch et al. 2016; Zhao et al. 2011). The *aromatic* subpopulation contains a *japonica* chloroplast genome and nuclear ancestry from both the *aus* and *temperate japonica* subpopulations, along with significant admixture from local wild ancestors (Civáň and Brown 2018).

Surprisingly, despite the relevance of root tissue to mineral nutrient acquisition and homeostasis, there are relatively few studies that attempt to survey the rice root ionome. Studies attempting to characterize the genetic architecture of root ion concentrations in rice have focused primarily on sodium (Na^+^) and potassium (K^+^) in the context of understanding the genetic mechanisms of salt tolerance (Lin et al. 2004; Patishtan et al. 2018; Wang et al. 2012; Zheng et al. 2015). Attempts to understand the genetic basis of the root ionome in other plant species have included studies in *Arabidopsis thaliana* (Ghandilyan et al. 2009a, b; Prinzenberg et al. 2010), *Thlaspi caerulescens,* a heavy metal hyper-accumulator (Assuncão et al. 2006; Deniau et al. 2006), barley (Nguyen et al. 2013), wheat (Hussain et al. 2017) and *Medicago truncatula* (Kang et al. 2019). While it has been reported in many species that genes expressed in root tissue often have dramatic influence on shoot and grain ionomic profiles (Byrt et al. 2014; Schaefer et al. 2018; Wu et al. 2019), it is interesting to note that in all of the aforementioned studies, there is virtually no overlap of genetic signal for the same element in both roots and above ground tissues. This lack of genetic correlation between tissues combined with the limited number of studies connecting quantitative variation for mineral ion concentration to natural genetic variation in rice roots means the genetic basis of the rice root ionome remains obscure. With almost half the world’s population depending on rice as a staple food, and increased focus on alternatives to the irrigated paddy system of rice production, understanding the acquisition, root–shoot partitioning, and processing of mineral nutrients in rice is of critical importance to the human diet.

To date, ionomic studies in rice have focused primarily on elemental accumulation in plant leaves and grain using either biparental QTL analysis (Norton et al. 2010a, b, 2012; Zhang et al. 2014) or single-marker genome-wide association analysis based on a variety of diversity panels (Li et al. 2013; Liu et al. 2020, 2019; Pinson et al. 2015; Yang et al. 2018; Zeng et al. 2010). Historical biparental QTL literature generally lacks genomic resolution (due to a paucity of markers) and thus defies physiological, molecular, or genetic characterization without further refinement. Combining legacy QTL information with more recent advances in genetic mapping permits the wealth of information collected from over a decade of genetic mapping research in rice to lend confidence to the higher resolution genetic signal detected in studies such as this one.

In addition, a wealth of candidate genes implicated in plant mineral homeostasis have been identified through electronic annotation, comparative genomics, and the integration of genome-wide association studies (GWA) with gene co-expression networks (Rampey et al. 2006; Schaefer et al. 2018; Whitt et al. 2020). These provide valuable sets of “known or predicted ionomic genes” (KIG) that can be used as *a priori* candidates associated with GWAS or biparental QTL studies across a range of plant species (Whitt et al. 2020).

In the present study, we applied a hydroponics-based phenotyping platform that allowed precise and accurate determination of ion concentrations for 20 elements of agronomic and/or biological importance in paired root and shoot samples from individual 6-week-old rice plants. Ionomic data was collected on a rice diversity panel (RDP1; Zhao et al. 2011) and a biparental recombinant inbred line (RIL) population (Azucena x IR64; Spindel et al. 2013; This et al. 2010) using inductively coupled plasma-optical emission spectroscopy (ICP-OES). Bayesian adjusted phenotypic line means were calculated and used for (a) single-marker GWA analysis, (b) multi-marker GWA using penalized orthogonal components regression (POCRE), and (c) composite interval mapping (in the RIL population) to identify genomic regions associated with each element in both roots and shoots from the same plants. The information was cross-referenced with a list of 321 a priori candidate genes implicated in the transport of mineral nutrients and with historical QTL data documented in the literature since 2005. Consistent with what has been observed in other species, a distinct portfolio of genomic regions and candidate genes were identified in root versus shoot tissue. This segregated pattern of genetic signal persisted even after partitioning the analysis to consider only distinct clades or subpopulations of rice. This study provides new information about the range of natural genetic variation available in the different subpopulations of *O. sativa*, the differences between the root and shoot ionomes, and provides a valuable baseline of information for future studies where element concentrations may be perturbed in an effort to understand the genetic basis of mineral nutrient homeostasis in domesticated Asian rice (*O. sativa*).

## Materials and methods

### Germplasm and plant growth conditions

**Fig. 1:**
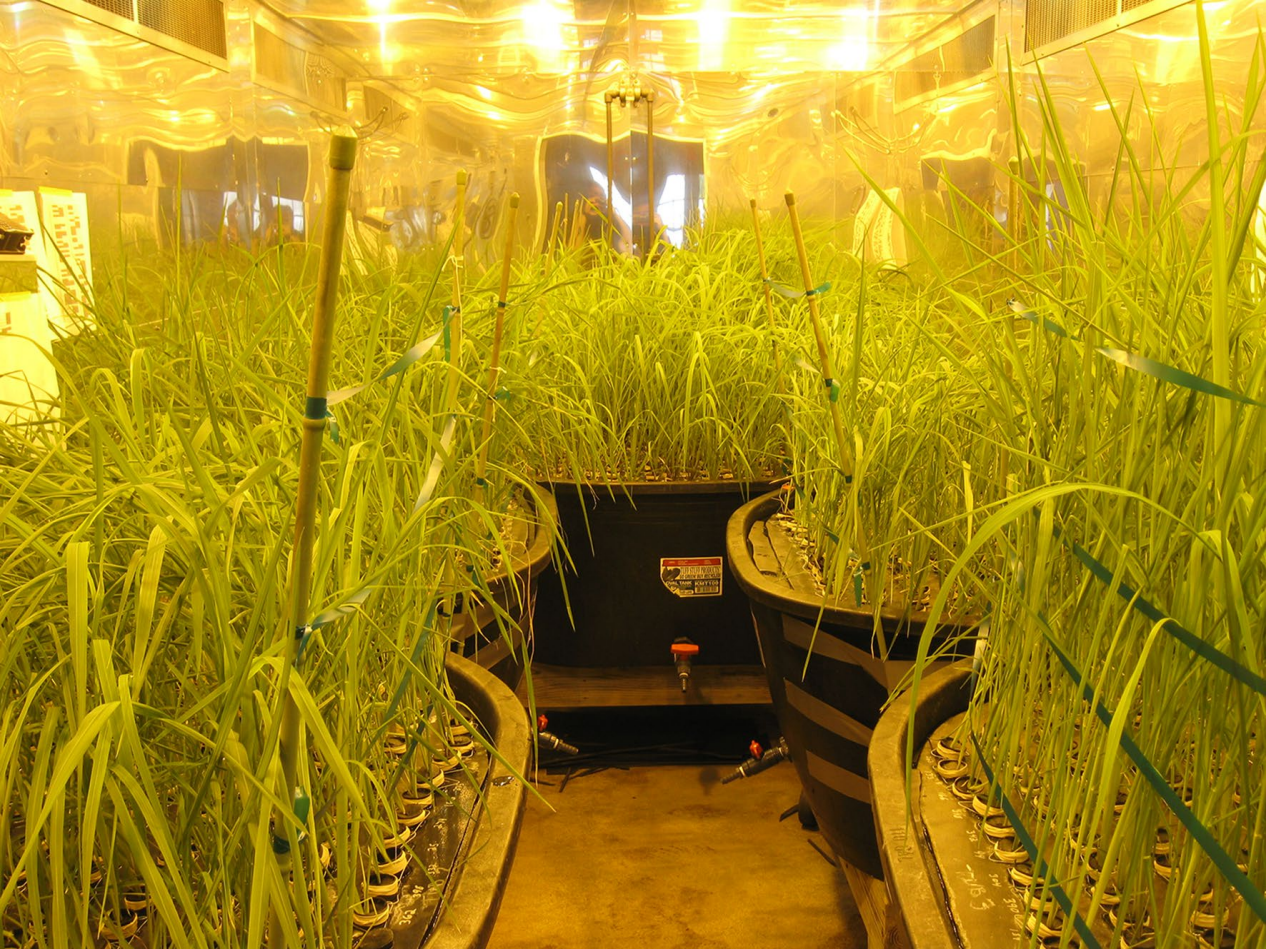
Six-week-old rice plants grown under hydroponics conditions. Rice plants grown from pre-germinated seedlings under hydroponic growth conditions to 6 weeks of age

The 373 genotypes used in the association analysis are part of a set of 410 *O. sativa* lines genotyped with the High-Density Rice Array (McCouch et al. 2016). Rice seeds were sterilized with 20% bleach for 15 to 20 min and germinated in rolled germination paper at 26 to 30 degrees C for 3–5 days under dark conditions. Upon germinating, seedlings were transferred to one of five 400 L hydroponics tubs by suspending them in foam circles embedded in modified 35 mm film canisters and placed in a foam framework that accommodated 200 plants per tub (Fig. 1). Individuals were randomized within and across tubs and seedlings were grown without aeration. Plants were stagger germinated such that five tubs were planted over the course of 5 days and likewise tissue was harvested over 5 days. The plants were grown in the hydroponic nutrient solution for 42 days before harvesting root and shoot tissue for ICP analysis. After the first 21 days, ribbon scaffolding was erected to support the shoots and prevent lodging.

At the time of harvesting, all of the accessions were in the vegetative growth stage and had an average root dry weight biomass between 150 and 200 mg, while the average shoot biomass had an average weight of 1.56 g. The amount of available tissue was critical, as precision estimates of trace elemental concentrations are often difficult to obtain when < 10 mg of dry weight tissue is used in ionomic analyses (Salt 2004). Using larger sample weights (particularly for root tissue) preserved the ability to precisely detect low levels of trace elements with the ICP-optical emission spectrometer, which depends on a minimum atomic signal from the sample.

The nutrient solution consisted of 1000 µM Ca(NO_3_)_2_·4H_2_O; 1000 µM NH_4_NO_3_; 250 µM KNO_3_; 250 µM KH_2_PO_4_; 250 µM MgSO_4_·7H_2_O; 1000 µM MES pH buffer; 77 µM Fe(NO_3_)_3_·9H_2_O; 77 µM H_3_EDTA; 300 µM NaOH; 50 µM KCl; 12.5 µM H_3_BO_3_; 0.1 µM Na_2_MoO_4_.2H_2_O; 2 µM Na_2_SeO_3_; 4 µM Na_2_HAsO_4_·7H_2_O; 0.1 µM KI; 0.1 µM K_2_Cr_2_O_7_; 0.1 µM NiSO_4_·6H_2_O; 2 µM MnSO_4_·H_2_O; 1 µM CuSO_4_·5H_2_O; 0.1 µM Co(NO_3_)_2_·6H_2_O; 2 µM ZnSO_4_·7H_2_O; 0.1 µM Cd(NO_3_)_2_·4H_2_O; 0.1 µM RbNO_3_; 0.1 µM Sr(NO_3_)_2_; 0.1 µM LiNO_3_; 0.1 µM PbNO_3_; and 145 µL/L of commercial AgSil 25 (http://www.pqcorp.com/) which contributed 600 µM K_2_SiO_3_. Chemical speciation was determined according to Shaff et al. (2010) using the GEOCHEM-EZ speciation program and the predicted ionic activity (availability) for each element was determined through the primary distribution and case progress table output. The solution in each tub was re-circulated daily with a magnetic drive pump and brought up to a pH of 6.0 using KOH. Samples of the nutrient solution were taken every other day and used to evaluate the nutrient composition of the media. Nutrients that depleted quickly were replenished as needed. The pH of the nutrient solution was maintained at 6.0 for the duration of the experiments. This differed from the normal pH of a rice paddy (typically 7.0) in order to (1) ameliorate the chemical sequestration of nutrients as the pH becomes more acidic over the course of 24 h due to ammonium uptake, (2) to ensure the general availability of all ions in the analysis, some of which lose availability at higher pH values, and (3) to reduce the amount of potassium hydroxide needed to maintain the pH of the nutrient solutions over time. The growing conditions were set to 30 °C daytime and 26 °C nighttime temperatures on a 12H light/dark cycle. Light intensity was approximately 450 mmol photons m^−2^ s^−1^.

At the time of harvesting, plant roots were desorbed in 5 mM CaCl_2_ for 15 min and then the entire plant was rinsed in deionized water prior to separating the roots from the shoots. Plant tissue was dried in a hot air drying oven at 60 °C for 5 days. The experiment was repeated twice (with two reps of each line each time) in order to compile the 4 reps that comprised the raw data that went into the Bayesian adjustment pipeline.

### ICP analysis for elemental composition

Dried shoot tissue was weighed, pulverized in liquid nitrogen, and 200 mg was subsampled for ICP analysis. As whole roots were analyzed, they were not pulverized prior to weighing and digestion. Each tissue sample was digested in a carbon heat block using a Vulcan 84 automated sample digestion unit (http://www.qtechcorp.com/). 250 µL of a 40 ppm Yttrium standard was added to each sample in order to back-calculate the appropriate dilution volume. Approximately 4 ml of 60/40 nitric/perchloric acid was added to each sample and heated to 150 °C. After incubating for an hour at 150 °C, the blocks were heated to 190 °C for 10 min. Samples were not allowed to digest to dryness and were subsequently diluted with deionized 18 MOhm water. ICP analysis was run on a Thermo 6000 series inductively coupled argon plasma-optical emission spectrometer housed at the US Department of Agriculture—ARS Robert W. Holley Center in Ithaca, NY.

Small variations in the delivery volume of the acid solution used to dilute the samples as well as evaporation after dilution has taken place impact the final dilution volume, and thus change the resulting concentration estimates. In an effort to improve dilution accuracy, a known volume and concentration of a yttrium standard was added to each sample. Yttrium is not absorbed by plant tissue, so is not natively present in the sample, but can be easily detected by the ICP. The yttrium concentration data detected by the ICP were compared to the expected concentration of yttrium that was added prior to the analysis and individualized dilution factors for every sample were determined rather than a standard dilution factor assumed across all samples. This had the added benefit of accounting for drift in the ICP measurements that inevitably occurs over runs with large sample numbers.

To extract robust estimates of line means for each genotype from the replicated measurements of ion concentration, we constructed a hierarchical Bayesian model (Gelman and Hill 2007). This model considers all phenotypes at once, estimates covariance among them, and takes into account the uncertainty in all parameters to construct marginal distributions of all variables of interest. For the purposes of this study, we are primarily interested in the portion of the phenotypic attribute of each rice line that is due to its genetic makeup (Greenberg et al. 2011). We assume that covariances come from inverse-Wishart distributions with vague diagonal priors (Greenberg et al. 2011). We have three levels of replication within which all the phenotypes are simultaneously modeled: 1) individual observations (with experimental errors, modeled as in Greenberg et al. (2011) are nested within lines, nested within 2) genome-estimated breeding values, with 3) an overall mean for the entire diversity panel as the final level. In addition, tub, batch, and experiment effects were not nested in the hierarchy and were modeled with a variable-intercept regression (Gelman and Hill 2007) at the level of individual observations. Genomic estimated breeding values are estimated using a Bayesian equivalent of the standard RR-BLUP model (Endelman 2011) using the Van Raden relationship matrix.

### Bayesian adjustment of phenotype data

Source code for the library used in the software that performed the model fit can be found at https://github.com/tonymugen/MuGen. Bayesian models were implemented using the open source statistical package R (https://www.r-project.org/). Replicates were modeled with a multivariate Student-*t* distribution with three degrees of freedom to modulate the influence of outlier observations. Multivariate Gaussian distributions were assumed for location parameters at the rest of the levels. Three technical replicates of each ion measurement were collected by the ICP for each sample. The Bayesian Relative Standard Deviation (RSD) was treated as a calculated measurement error from these technical replicates and used to weigh individual observations within the hierarchical model, as described by Greenberg et al. (2011). We noted four sets of replicate-level conditions with the potential to affect systematically our observations: the position of each plant within the growth chamber, the tub identity, the identity of the individual harvesting the tissue, and the identity of the digestion batch as each sample was prepared for ICP analysis. We constructed dummy variables reflecting contrasts within each set of covariates, but some of the resulting contrasts were collinear. To circumvent this problem, we estimated principal components of the corresponding covariance matrix and used principal component vectors that corresponded to nonzero eigenvalues as predictors in a variable-intercept regression at the replicate level in our model. To obtain biologically interpretable data-points, the levels of each element in each sample were divided by the weight of that sample. While this procedure of dividing by sample weight is preferable in highly structured experiments such as ours, because whole roots (rather than subsamples) were analyzed due to their smaller size, results have to be interpreted with the possibility of spurious correlations among variables in mind, particularly for the root samples. Only a portion of each shoot was used, resulting in a very narrow distribution of sample weights. A detailed discussion of the pitfalls and advantages of per-weight measurements can be found in Greenberg et al. (2011) The phenotypic means used for GWAS and QTL analysis can be accessed from our project web site: see https://www.zstats.org/rice/index.html.

### Genotype data and preprocessing

Genotype data from the High-Density Rice Array (HDRA) were downloaded from: http://www.ncbi.nlm.nih.gov/geo/query/acc.cgi?acc=GSE71553. It includes 1,568 samples genotyped with 700,000 SNPs. After matching genotype data with phenotype data based on the sample ID, we used PLINK (v1.07; Purcell et al. 2007) to filter out SNPs with missingness per marker > 0.3, and minor allele count (MAC) < 6 for subpopulation analyses, and MAC ≤ 10 for analyses involving ALL, INDICA and JAPONICA. All missing data for a given genotype were imputed with the sample mean of the respective variable.

After matching the genotype data with the phenotype data, 373 samples were used for single- and multi-marker analysis among ALL subpopulations, 131 samples for the INDICA clade, 223 for the JAPONICA clade, as well as 53 for *aus,* 72 for *indica,* 92 for *tropical japonica,* and 104 for *temperate japonica* subpopulations (summarized in Suppl. Table S-5).

### Genome-wide association analysis

#### Principal component analysis

We calculated the first three principal components of the genotype data in ALL, INDICA, and JAPONICA using MATLAB (R2014a) and used the PCs as covariates in the linear regression of the ionomic phenotypes, and the residuals from this regression were used for single-marker and multi-marker analysis, as described below.

#### Single-marker analysis

Efficient Mixed Model Association eXpedited (EMMAx) (Kang et al. 2010) was used to run single-marker analysis (SMA) for each root and shoot ionomic trait. The response variable for each regression model is the residual value of each root and shoot trait, and the predictor is the genotype of each SNP. For analyses involving the entire RDP1 (ALL, *n* = 373), the first three PCs were used; for analyses using the INDICA and JAPONICA clades, the first PC only was used; for analyses involving each subpopulation individually (*aus, indica, tropical japonica, temperate japonica*), no PCs were used. A “GWA peak” was defined by the presence of ≥ 3 significant SNPs within a 200 kb interval, where each SNP has a *p* value < 1 × 10^–5^ and individual GWA peaks were separated from each other by > 200 kb. Results of all SMA for ion/tissue/germplasm group combinations can be accessed via the project web site: http://www.zstats.org/rice/index.html.

#### Multi-marker analysis

The penalized orthogonal components regression (POCRE) (Zhang et al. 2009a, b) algorithm was used to run multi-marker analysis (MMA) for each root and shoot trait. The response variable for each regression model is the residual value of each root and shoot trait (as described above) and the predictors are the chromosome-wise genotypes. After standardization, POCRE sequentially constructs orthogonal components by finding penalized leading principal components, allowing us to effectively identify sparse predictors of each component among a huge number of SNPs across the entire chromosome. The nonzero coefficient associated with each SNP is employed to determine the significance along with the results from SMA and other information.

### Biparental (IR64 x Azucena) QTL mapping

Ionomics phenotyping was done using the hydroponics protocol described above with 145 lines from a previously described recombinant inbred line population and previously generated genotyping-by-sequencing SNP data (Spindel et al. 2013; This et al. 2010). Phenotypic means were estimated using the Bayesian adjustments described above. SNPs with greater than 15% missing data were excluded as were individuals from the population with greater than 15% missing genotype data. Composite interval mapping was done using the R/QTL package (Broman et al. 2003).

### Candidate genes used for annotating GWA peaks

A list of 321 a priori candidates was assembled using genes identified in the literature as involved in the uptake, homeostasis, and regulatory processes related to mineral nutrition or toxicity (Suppl. Table S-4). Genes identified in rice, as well as the rice homologs of genes identified in other species, were included. The list is comprised mostly of membrane transporters, plus a smaller proportion of transcription factor genes, and contains 321 unique gene models. All known members of membrane transporter families involved in ion uptake were included, whether or not they have been characterized.

### Haplotype analysis

Using the imputed SNP dataset for the rice diversity panel (Wang et al., 2018), SNPs were filtered to remove those with minor allele count ≤ 6. This procedure was done sequentially using the full dataset (ALL, n = 373 accessions), the two clades (INDICA and JAPONICA) and each subpopulation (*aromatic, aus, indica, tropical japonica, temperate japonica),* with admixed lines excluded from the subpopulation-specific analyses, as described in McCouch et al. (2016). The set of SNPs used to construct haplotypes across each region of interest included (a) the most significant SNPs associated with the phenotype, (b) SNPs mapping in candidate genes of interest, and (c) a subset of SNPs that distinguished each of the haplotypes across the region of interest, eliminating those in perfect LD.

## Results

### Phenotyping and heritability

Three hundred seventy-three inbred varieties of *O. sativa* representing the Rice Diversity Panel 1 (RDP1) (Eizenga et al. 2013; Zhao et al. 2011) were grown hydroponically to 6 weeks of age (Fig. 1) and evaluated for ion concentrations in root and shoot tissue. A standard nutrient solution was used to provide all of the macro- and micronutrients necessary for plant growth and modified to include sub-toxic levels of heavy metals and other ions of biological or agronomic interest (see Materials and Methods). The list of 20 elements included in the study, lower limit of detection (in ppm) of each element, Bayesian relative standard deviation (RSD; see Materials and Methods) of three technical replicates run on each sample, and the broad-sense heritability (H^2^) of ionomic phenotypes (0.10–0.56) are summarized in Table 1 (see also Suppl. Figure S-1). Estimates of potassium (K) and lead (Pb) were reliable in roots but not in shoots, so were excluded from further analysis in shoots.

**Table 1:** Precision and accuracy of detection and heritability (H^2^) of 20 root and 18 shoot ionomic phenotypes measured on the Rice Diversity Panel 1 (n = 373)

Abbrev	Ion	Lower limit of detection (ppm)	Bayesian RSD*		Heritability (H^2^)	
			Roots	Shoots	Roots	Shoots
As	Arsenic	0.038	16.89	34.03	0.14	0.13
B	Boron	0.146	36.18	9.97	0.17	0.18
Ca	Calcium	0.291	10.32	11.54	0.18	0.31
Cd	Cadmium	0.004	17.20	22.72	0.28	0.33
Co	Cobalt	0.003	15.15	26.01	0.22	0.12
Cr	Chromium	0.022	18.34	6.11	0.14	0.15
Cu	Copper	0.005	24.28	18.85	0.30	0.14
Fe	Iron	0.240	15.99	16.96	0.16	0.25
K	Potassium	6.864	13.71	NA	0.24	NA
Mg	Magnesium	1.219	13.81	10.43	0.30	0.38
Mn	Manganese	0.001	24.23	21.34	0.20	0.25
Mo	Molybdenum	0.003	68.87	13.76	0.43	0.56
Na	Sodium	0.788	15.86	18.00	0.16	0.21
P	Phosphorus	2.794	9.73	8.15	0.29	0.40
Pb	Lead	0.008	22.16	NA	0.10	NA
S	Sulfur	0.509	9.79	9.71	0.46	0.37
Se	Selenium	0.014	11.92	10.43	0.32	0.28
Si	Silicon	0.089	22.89	14.30	0.11	0.22
Sr	Strontium	0.001	14.32	10.86	0.18	0.34
Zn	Zinc	0.065	18.31	18.63	0.24	0.11

Heritability estimates represent Bayesian approximations of broad-sense heritability (H^2 = Var(among lines)/[Var(among lines) + Var(among replicates)]) of shoot and root phenotypes

NA not included in GWAS due to high Bayesian RSD and/or low H^2^

*RSD = Bayesian relative standard deviation

### Single-marker GWA

**Fig. 2:**
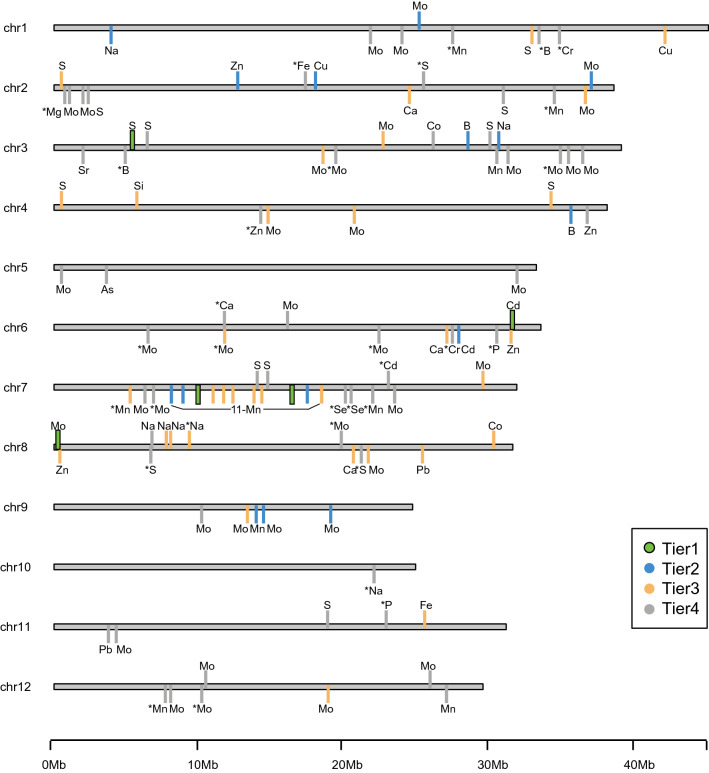
Graphical representation of significant genome-wide associations. Bright green bars = Tier 1 peaks; blue bars = Tier 2 peaks; orange bars = Tier 3 peaks; gray bars = Tier 4 peaks; bars above chromosomes = shoot associations; bars below chromosomes = root associations; elements are abbreviated as in Table 1; asterisks = associations detected uniquely in subpopulations

A total of 114 GWA peaks associated with 19 elements were identified by single-marker analysis (SMA) using Efficient Mixed Model Association eXpedited (EMMAx) (Kang et al. 2010) and a genotyping dataset consisting of 700,000 SNPs (McCouch et al. 2016) (Fig. 2; Suppl. Table S-2; Suppl. Table S-1). A GWA “peak” was defined by the presence of ≥ 3 significant SNPs (*p* value < 1 × 10^–5^) located within ≤ 200 kb of each other (see Materials and Methods). Of the 114 peaks identified, 78 were identified in root tissue, and 36 in shoots (Table 2). Peaks associated with root and shoot tissue were sometimes linked (i.e., Mo on chromosome 2 and 12), but in no case were identical peaks identified for the same element in both tissues (Fig. 2).

**Table 2:** Summary of GWAS peaks detected in ALL, Clades, and Subpopulations

Populations	Unique Peaks		TOTAL
	Root	Shoot	Root + Shoot
**ALL + Clades**			
ALL	34	20	54
ALL/*JAPONICA*	11	3	14
ALL/*INDICA*	1	0	1
ALL/*JAPONICA/INDICA*	0	1	1
* JAPONICA*	6	3	9
* INDICA*	2	2	4
Sub-total unique peaks	54	29	83
Subpopulations*			
*aus*	9	2	11
*indica*	4	2	6
* tropical japonica*	7	1	8
* temperate japonica*	4	2	6
Sub-total unique peaks	24	7	31
Grand Total Unique Peaks	78	36	114

*Subpopulation peaks shown here represent only those uniquely detected by subpopulation-specific analyses; in addition, 20 of the peaks reported for ALL + Clades were supported by subpopulation peaks (*aus* = 5; *indica* = 3; *tropical japonica* = 11; *temperate japonica* = 1), as detailed in Suppl. Table S-1 and Suppl. Table S-2

**Fig. 3:**
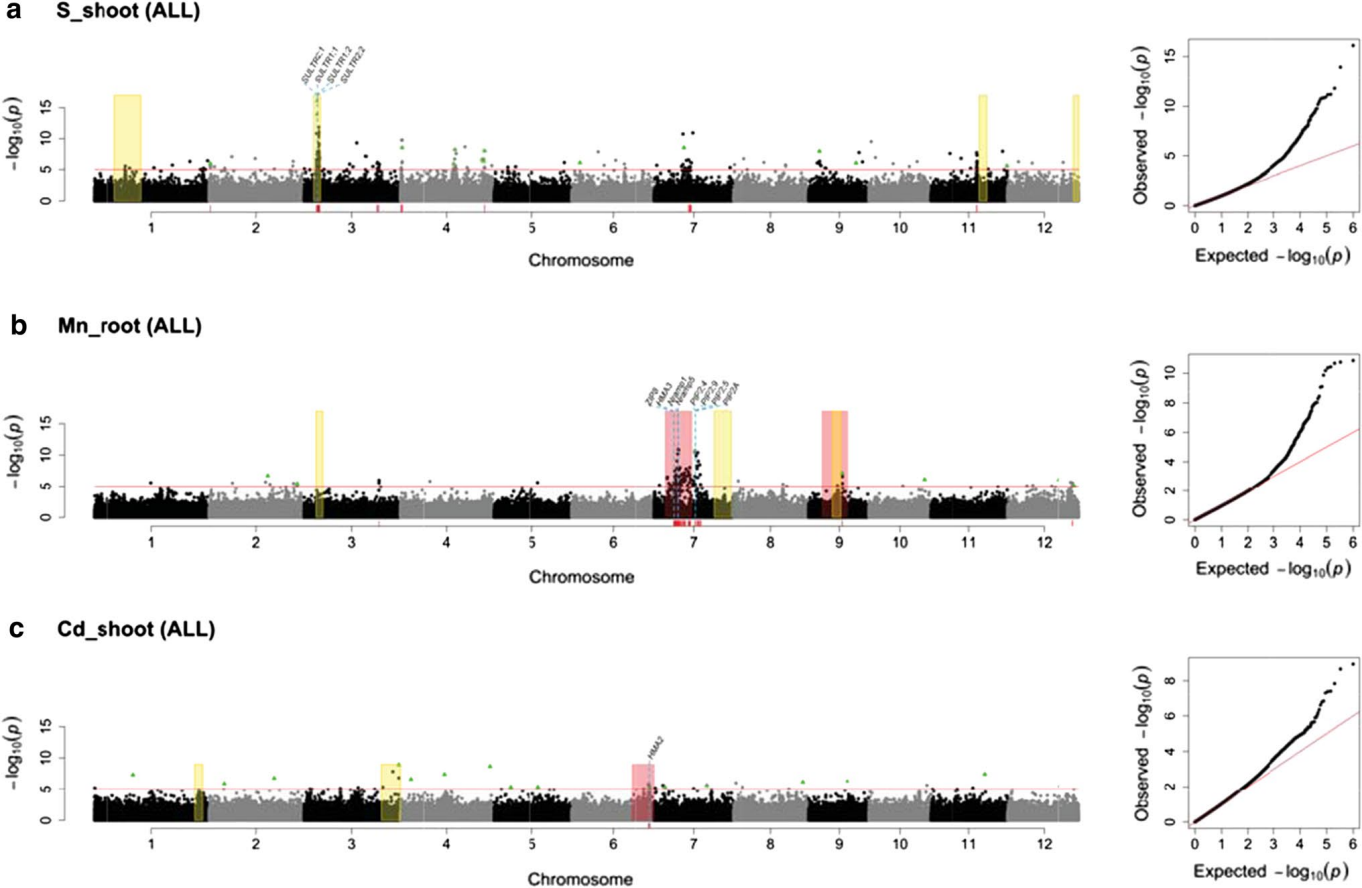
Selection of integrated genome-wide Manhattan and QQ plots for **a** S_shoot in the complete panel (ALL), **b** Mn_root (ALL), and **c** Cd_shoot (ALL). Similar plots for all phenotypes, clades, and subpopulations can be found at https://www.zstats.org/rice/indec.html. Yellow boxes indicate the presence of QTL detected using the same phenotyping protocol in the Azucena x IR64 RIL population (see Supplementary Table S-3; box width scaled to significant interval for each QTL); pink boxes indicate the presence of a previously published legacy QTL (see Supplementary Table S-6; box widths standardized to 10 Mb surrounding the reported peak marker); red markers along the chromosomal axis indicate called GWA peaks (identified in Supplementary Table S-1); green triangles indicate SNPs identified as significant by POCRE analysis. Candidate genes are indicated by their respective gene names and a blue dashed line indicating the genomic position of their midpoint

GWA was first performed using the entire rice diversity panel (ALL, n = 373) and subsets representing the JAPONICA (*n* = 220) and INDICA (*n* = 131) clades to gain insight into the origins of the alleles driving the associations (McCouch et al. 2016). Manhattan and QQ plots summarizing the analysis of each combination of element/tissue/germplasm group (Fig. 3) can be accessed on our web site (https://www.zstats.org/rice/index.html). Of the 114 total GWA peaks reported in this study, 83 were identified in these analyses; nine peaks were uniquely detected in the JAPONICA clade, four uniquely in the INDICA clade, and 54 uniquely using ALL, with an additional 16 peaks detected in one or both clades in addition to ALL. These peaks were associated with 15 elements, as summarized in Suppl. Table S-2. Using only the JAPONICA clade, 24 peaks associated with eight elements were identified, and using INDICA, six peaks associated with six different elements were identified. Peaks associated with Ca were detected uniquely in the JAPONICA clade while peaks associated with As, Fe, Si, and Sr were detected uniquely in INDICA. In only one case was a peak identified in ALL, JAPONICA, and INDICA (Peak #48 on chromosome 8 for Mo_shoot) (Suppl. Table S-2; Suppl. Table S-1).

GWA was additionally performed using the four major subpopulations that comprise the clades: *tropical japonica* and *temperate japonica* for the JAPONICA clade, *aus*, and *indica* for the INDICA clade (Table 2; Suppl. Table S-2; Suppl. Table S-1)*.* Though sample sizes were small, the subpopulations are well differentiated in rice, and these analyses provided an opportunity to investigate putative associations between ionomic phenotypes and SNPs that are common in one subpopulation but rare in the panel as a whole. A total of 51 peaks were identified in the subpopulation-specific analyses, of which 31 were new and 20 were co-located, providing additional support for peaks previously identified for the same element and tissue in INDICA, JAPONICA, and/or ALL (Suppl. Table S-2). For example, multiple peaks associated with Mn_root were detected across a broad region on chromosome 7 in the *tropical japonica* (but not the *temperate japonica*) subpopulation, and the same peaks were also detected in ALL and in JAPONICA, supporting the hypothesis that the variation driving these associations at the panel or clade levels derives from the *tropical japonica* gene pool. There were no INDICA peaks for Mn_root in this region (nor peaks detected in the *aus* or *indica* subpopulations). In other cases, GWA peaks were identified in one of the subpopulations and its corresponding clade, but not in ALL (i.e., peak #78 in INDICA and *aus* for Si_shoot on chromosome 4, and peak #13 in INDICA and *indica* for Fe_shoot on chromosome 11). Interestingly, subpopulation-specific analyses revealed peaks associated with Cr, Mg, P, and Se for the first time (Suppl. Table S-2; Suppl. Table S-1).

Of the 20 elements investigated here, the largest number of peaks was discovered for molybdenum (Mo) (30 in roots, 9 in shoots), followed by manganese (Mn) (19 in roots, none in shoots) and sulfur (S) (5 in roots, 10 in shoots), with seven or fewer peaks identified for each of the other elements (Suppl. Table S-2). GWA peaks were distributed on all 12 chromosomes, with a large cluster associated with Mn on the long arm of chromosome 7, and only one peak (peak #106, Na_root detected in *tropical japonica*) on chromosome 10 (Fig. 2).

### Multi-marker GWA

In addition to single-marker GWA analysis, we employed a variable selection approach, referred to as penalized orthogonal components regression (POCRE; Zhang et al. 2009b). First, we simultaneously screened chromosome-wise SNPs and selected a subset of candidate SNPs identified by POCRE. Next, we identified the intersection of POCRE SNPs and significant SMA SNPs (*p* value < 10^–5^) and determined which of the intersection set mapped within the GWA peaks. The convergence of POCRE and significant SMA SNPs was used to increase the confidence level of the GWA peaks in ALL, INDICA, and JAPONICA. Of the 83 GWAS peaks identified by SMA, 33 (40%) were supported by POCRE. The supported peaks were identified in both root and shoot tissue and were associated with 13 elements (Suppl. Table S-1; Manhattan plots available at https://www.zstats.org/rice/index.html).

### Biparental QTLs detected in the IR64 x Azucena RIL population

A biparental population consisting of 145 recombinant inbred lines (RILs) derived from a cross between IR64 (*indica)* x Azucena (*tropical japonica*) and previously genotyped with 30,982 SNP markers (Spindel et al. 2013) was phenotyped following the same hydroponic protocol used with RDP1. A set of 106 QTLs associated with 19 elements were detected in root and shoot tissue of the RILs (Suppl. Figure S-2; Suppl. Table S-3). QTLs were distributed on all chromosomes except for chromosome 10, and the average interval size of a biparental QTL was 3.66 Mb (18 × the size of the average GWA peak). Six (5.7%) of the biparental QTLs overlapped with GWA peaks for the same element, and four had additional support from POCRE SNPs (Suppl. Table S-1).

As with the GWA peaks, overlapping QTLs associated with the same element in different tissues (roots and shoots) were also rare in the RIL population, with Mo and P being the only exceptions. Mo_root and Mo_shoot QTLs were found co-located on chromosomes 1 and 7, and P_root and P_shoot QTLs overlapped on chromosome 4 (Suppl. Figure S-2; Suppl. Table S-3).

There were also examples where GWA signal for one tissue type overlapped with biparental mapping signal for the other tissue type for the same element. Two regions exhibiting this kind of overlap were of particular interest, one on chromosome 2 where closely linked GWA peaks (peaks #33 and #34) for Mo_shoot (160 kb) and Mo_root (168 kb) overlapped with a single, large (3 Mb) biparental QTL for Mo_root, and one on the long arm of chromosome 7 where a cluster of GWA peaks for Mn_root (peaks #23, #24, and #25) overlapped with a 4.5 Mb QTL associated with Mn shoot in the RIL population (Suppl. Table S-1; highlighted in yellow in the Manhattan plots at https://www.zstats.org/rice/index.html). Both have support from POCRE SNPs.

### Legacy QTL

A literature survey was undertaken to identify previously reported biparental and GWA QTLs associated with the ions for which signal was detected in this study (GeneFlow database). A total of 22 QTLs associated with nine different elements reported in 20 studies published between 2005 and 2020 co-localized with unique GWA peaks identified in this study (Suppl. Table S-1 and Suppl. Table S-6). Most previous studies had very poor resolution due to the paucity of markers available at the time of publication (average QTL size was ~ 10 Mb), but careful curation of SSR and RFLP marker positions enabled their placement on the rice genome (IRGSPv1/ MSUv7). These QTLs were found on 11 of the 12 chromosomes (with the exception of chromosome 5) and all mapped within 200 kb of a significant SNP detected by our GWA results for the same element (Suppl. Table S-1; Regions highlighted in pink in Manhattan plots available at https://www.zstats.org/rice/index.html).

### Co-location of GWA peaks with legacy QTL from field studies

Three previous GWA studies identified ionomic QTLs from leaf and/or grain samples using field-grown rice diversity panels (Liu et al. 2019; Norton et al. 2014; Yang et al. 2018). These studies focused on sixteen (Liu et al. 2019; Yang et al. 2018) and four (Norton et al. 2014) of the same elements as the current study. When positions of their GWA signals were compared with those reported in this study, twelve were found for the same elements in overlapping genomic locations (one for Cd_shoot, two for Mn_root, four for Mo_shoot, and five for Mo_root), often in differing tissue types (Suppl. Table S-1; Suppl. Table S-6). Among biparental QTL studies, 14 distinct hydroponically derived GWA peaks from our study were detected as overlapping with QTL previously mapped in field-grown above ground tissue for the same element for the following traits: B (root and shoot) Cd (root and shoot) Cu (root and shoot) Fe_shoot, Mn_root, Mo (root and shoot), Na (root and shoot) and Zn_shoot (Suppl. Table S-1 and Suppl. Table S-6).

### Candidate gene analysis

A list of 321 a priori candidate genes associated with ionomic phenotypes (Supplementary Table S-4) was compiled using gene ontology (GO) terms related to the transport and movement of ions across plant cell membranes and throughout the plant body, and augmented by lists compiled by other authors (Whitt et al. 2020; Yang et al. 2018). When comparing the location of these genes with GWA peaks in this study, a total of 14 *a priori* candidate genes were located within five GWA peaks, associated with four different elements: Cd, Mn, Mo, and S (Supplementary Table S-1). The five peaks containing candidate genes were further supported by POCRE SNPs, QTLs, or both.

### Tier classification

The 114 unique GWA peaks identified across elements, tissues, clades, and subpopulations were classified into four tiers, based on the amount of supporting evidence for each (Fig. 2). These classifications provide a way to prioritize GWA peaks for additional functional genomics research. Peaks containing candidate genes that were also supported by both POCRE SNPs and QTLs detected in the IR64 x Azucena RIL population and/or as legacy QTLs in the literature were classified as Tier 1 peaks; five peaks met these criteria. Fifteen peaks were supported by POCRE SNPs and QTLs, but not candidate genes, and were classified as Tier 2, 34 peaks were supported by either POCRE SNPs or QTLs but not both, and were considered as Tier 3, and 60 peaks were classified as Tier 4 because they were present only in the SMA analysis and not supported by POCRE SNPs, QTLs or candidate genes (Suppl. Table S-1).

### Genetic dissection of Tier 1 peaks

The five Tier 1 GWA peaks are associated with (a) sulfur in shoots (S_shoot) on chromosome 3, (b) two linked peaks for manganese in roots (Mn_root) on chromosome 7, (c) cadmium in shoots (Cd_shoot) on chromosome 6, and (d) molybdenum in shoots (Mo_shoot) on chromosome 8 (Suppl. Table S-1; Fig. 2). These regions were originally identified as SMA peaks using the HDRA SNP dataset with highly significant *p* values, and the candidate genes identified within these regions provided targets for further genetic dissection. To improve resolution of the analyses, we leveraged genotype data imputed from the 700 k HDRA SNP dataset using the methodology described in Wang et al. (2018). The imputed dataset, referred to as RICE-RP, consisted of 4.8 M genome-wide SNPs and provided an opportunity to focus on chromosomes and chromosomal regions carrying Tier 1 GWA peaks to examine the haplotype structure corresponding to the different clades and subpopulations of rice. The information is summarized below and provides the foundation for postulating likely sources of functional variation underlying these associations.

### Shoot sulfur

**Fig. 4:**
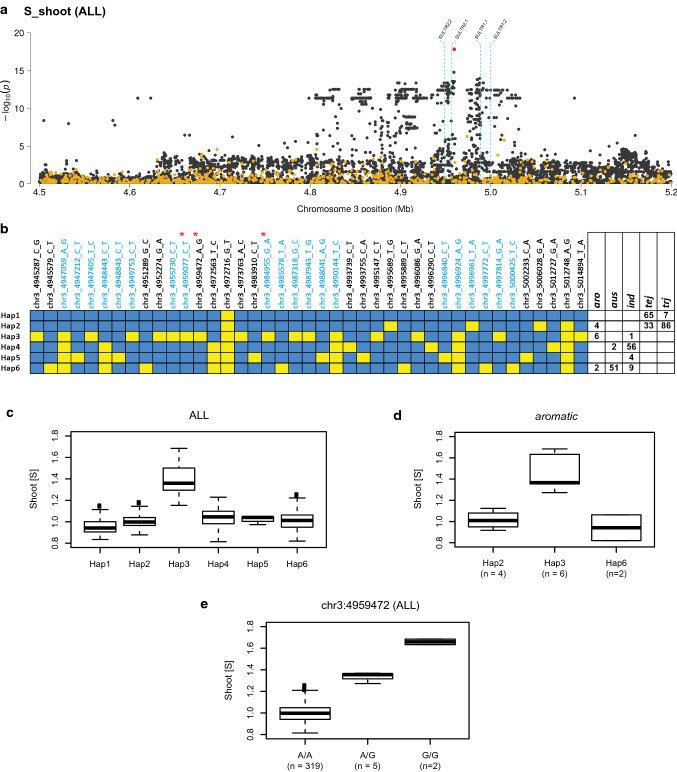
A region associated with S_shoot that co-localizes with a cluster of sulfate transporter genes on rice chromosome 3. **a** Zoom-in of chromosome 3 (4.5–5.2 Mb) showing GWAS peak in ALL; dotted blue lines indicate positions of candidate genes; gold dots represent SNP p values from single-marker GWAS using the unimputed HDRA SNP dataset; black dots represent SNP p values from chromosome-specific analysis using the imputed SNP dataset; red dot represents the MS-SNP using imputed genotype data.** b** Haplotype analysis of the 70 kilobase region surrounding four candidate genes. Blue boxes = reference (Nipponbare) alleles; yellow boxes = alternate alleles; SNPs labeled in blue map within candidate genes; red asterisks indicate the three MS-SNPs in single-marker analysis; boxes to the right indicate number of lines carrying each haplotype within a subpopulation. **c** Quantile boxplots show phenotypic distribution of S_shoot content in haplotype groups in ALL. **d** Quantile boxplots show phenotypic distribution of S_shoot in haplotype groups found in *aromatic* subpopulation. **e** Quantile boxplots show phenotypic distribution of S_shoot content across genotypic classes detected by MS-SNP (SNP-3:4,959,472); for all boxplots, edges represent the upper and lower quartile with median value shown as a bold line; whiskers represent 1.5 ×  the quantile of the data; individuals falling outside the range of the whiskers shown as dots

A significant GWA association for shoot sulfur concentration (S_shoot; peak #70 in Suppl. Table S-1) on chromosome 3 was detected in ALL, supported by two POCRE SNPs, an IR64xAzucena QTL (#91; Suppl. Table S-3), and a cluster of four SULTR transporter genes: *OsSULTR1;1*, *OsSULTR1;2*, *OsSULT1*, and *OsSULTR2;2* (Fig. 4a; Manhattan Plot for S_shoot at https://www.zstats.org/rice/index.html).

When high-resolution GWA was conducted using the imputed HDRA-RP dataset for chromosome 3, the MS-SNP (SNP-3:4,959,472) mapped in the middle of the gene cluster, 24 bp downstream of the *SULTR2;1* gene model (highlighted as red dot in Fig. 4a). The second most significant SNP (SNP-3:4,959,077) mapped 395 bp upstream within exon 1 of the *SULTR2;1* gene and the third MS SNP (SNP 3:4,984,955) mapped within exon 1 of the *SULTR1;1* gene (Fig. 4a; red asterisks in Fig. 4b). Based on this finding, we focused further genetic dissection and haplotype analysis on the interval spanning the four *SULTR* genes (chr3:4,945,287–5,014,894 bp). Haplotype analysis across this ~ 70 kb region revealed six well-differentiated regional haplotypes (Fig. 4b). Haplotypes 1 and 2 (Hap1 and Hap2) were found at high frequency in the *temperate japonica* and *tropical japonica* subpopulations, Hap3 was predominant in *aromatic,* Hap4 and Hap5 in *indica,* and Hap6 was at highest frequency in *aus.* We next examined the relationship between haplotype groups and the S_shoot phenotype and discovered that lines carrying Hap3 had significantly higher S_shoot than lines carrying any of the other haplotypes (Fig. 4c).

Overall, Hap3 is rare but it has a large phenotypic effect that drives the significance of the peak detected in ALL. The most significant SNPs across the region are all in LD and collectively define Hap3, which is carried by 50% of the *aromatic* accessions. The *aromatic* lines that do not carry Hap3 have either the *temperate japonica* like Hap2 (*n* = 4) or the *aus* like Hap 6 (*n* = 2) across this region and these lines have low S_shoot (Fig. 4d**),** consistent with observations shown in Fig. 4c**.** Six of the seven lines carrying Hap3 are classified as *aromatic* and one as *indica.* The single *indica* line with this haplotype is a variety from Myanmar (EMATA A 16–34) that exhibits 26% higher S_shoot than other *indicas*.

We next looked at the genotype data for the MS-SNP (SNP-3:4,959,472) located 24 bp downstream of the *SULTR2;1* gene and found that it was heterozygous in five of the seven lines that carried Hap3. When S_shoot is compared in lines carrying the A/A, G/G, or A/G genotypes, we observed a significant co-dominant effect at this SNP (Fig. 4e). Taken together, these data suggest that a rare haplotype harboring a cluster of *SULTR* gene family members and found at high frequency in the *aromatic* subpopulation is responsible for high S_shoot among these *O. sativa* lines*.*

### Root manganese

**Fig. 5:**
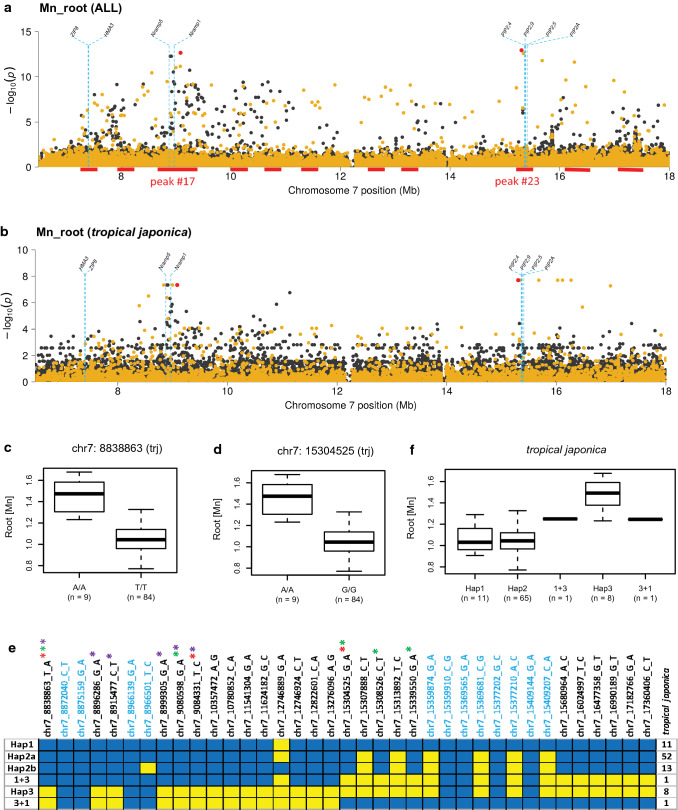
Extended region on rice chromosome 7 associated with Mn_root concentration co-localizing with a priori candidate genes.** a** Zoom-in of chromosome 7 (6.0–18.0 Mb) showing GWA peaks in ALL; dotted blue lines indicate position of candidate genes; gold dots represent SNP p values from GWAS using the unimputed HDRA SNP dataset; black dots represent SNP p values from chromosome-specific analysis using the imputed SNP dataset; red dots represent MS-SNPs using imputed genotype data. Red bars underneath the plot indicate GWA peaks as reported in Supplementary Table S-1. **b** Zoom-in of same region on chromosome 7 showing GWA peaks in *tropical japonica* subpopulation. **c** and **d** Phenotypic differences for Mn_root associated with MS-SNPs highlighted in red in peaks #17 and #23, respectively. **e** Haplotype analysis of 8.5 Mb region extending across peaks #17 and #23; blue boxes indicate reference (Nipponbare) alleles; yellow boxes indicate alternate alleles; SNPs labeled in blue map within Nramp or PIP gene clusters; red asterisks indicate MS-SNPs. Boxes to right indicate number of lines carrying each haplotype within *tropical japonica*. **f** Quantile boxplots show phenotypic distribution of Mn_root content in haplotype groups found in *tropical japonica*; for all boxplots, edges represent the upper and lower quartile with median value shown as a bold line; whiskers represent 1.5 × the quantile of the data; individuals falling outside the range of the whiskers shown as dots

A cluster of 11 closely linked GWA peaks for Mn_root in ALL was found across a large region (> 7.5 Mb) on chromosome 7 (Fig. 5a; peaks #15—#25 in Suppl. Table S-1). A majority of the peaks were also associated with Mn_root in the JAPONICA clade and in the *tropical japonica* subpopulation. A QTL identified by Yang et al. (2018) for Mn concentration in above ground biomass of field-grown plants overlapped with peaks #15 and #17, and a QTL detected in the IR64 x Azucena RIL population in our study for Mn_shoot overlapped with peaks #23, #24, and #25 (Suppl. Figure S-2; #61 in Suppl. Table S-3; Manhattan Plots on our web site: https://www.zstats.org/rice/index.html).

Two of the Mn_root peaks (Peaks #17 and #23) were classified as Tier 1 peaks; both were supported by POCRE SNPs, overlapping QTLs, and candidate genes from our *a priori* list, and both were identified in *tropical japonica,* JAPONICA, and in ALL (Fig. 5a). Peak #17 contained two candidate genes: *OsNramp1* and *OsNramp5* and the MS-SNP (SNP-7:9,084,331) mapped 113.5 kb downstream of the *OsNRAMP1* gene **(**Fig. 5a and e). The region corresponding to peak #23 overlapped with a cluster of four aquaporin transporters in the PIP2 family (*OsPIP2;4*, *OsPIP2;5*, *OsPIP2;9*, and *OsPIP2A*) and the MS-SNP (SNP-7.15304525, also identified by POCRE) mapped 54 kb upstream of *PIP2;4* **(**Fig. 5a and e).

We next examined the association between SNPs from the imputed dataset and the Mn_root phenotype in the *tropical japonica* subpopulation, and again, peaks #17 and #23 were highly significant (Fig. 5b). Phenotypic differences associated with the MS-SNPs in both the *Nramp* and the *PIP* gene regions in the *tropical japonica* analysis are shown in Fig. 5c and 5d. To examine the patterns of variation across the region spanning peaks #17–#23, we generated haplotype graphs as shown in Fig. 5e. Three major haplotype groups were observed within *tropical japonica,* along with a low frequency of recombinant haplotypes. Hap1 (*n* = 11), Hap2a (*n* = 52) and Hap2b (*n* = 13) were shared with *temperate japonica* lines, and Hap3 (*n* = 8) was private to a small group of *tropical japonica* lines. Hap3 carried alternate alleles at the MS-SNPs across both the *Nramp* gene region and the PIP gene regions (yellow highlighted alleles in Fig. 5e). When Mn_root was compared among the haplotype groups in *tropical japonica*, lines carrying Hap 3 had consistently higher Mn_root than lines carrying either Hap1 or Hap2 (Fig. 5f**).**

Two of the *tropical japonica* accessions contained recombinant haplotypes between Hap3 and Hap1**.** One carried Hap3 across the *Nramp* gene region and Hap1 across the *PIP* gene region (3 + 1 in Fig. 5e), and the other carried the reciprocal recombination event (1 + 3 in Fig. 5e)**.** To determine whether these recombinants were informative in assessing the relative phenotypic contribution of the *Nramp* and the *PIP* gene regions, we compared Mn-root concentration in the two recombinant lines and compared it with mean Mn_root concentration in lines carrying Hap3 across the entire region. The two recombinant lines both had mean Mn_root concentrations of 1.25 ppm, while Hap3-containing lines had a mean Mn_root value of 1.48 ppm (Fig. 5f). Further analysis of variation within the *PIP* gene models indicated that lines carrying Hap2a and Hap2b carry the same genic variants as lines carrying Hap3, while differing in their patterns of variation upstream and downstream of the *PIP* genes (Fig. 5e).

### Shoot cadmium

Similar to S_shoot and Mn_root, a Tier 1 peak for Cd_shoot was detected on chromosome 6 (Peak #8 in Suppl. Table S-1) in ALL and in the *tropical japonica* subpopulation (Suppl. Figure S-3a and b). It co-localizes with two POCRE SNPs, three legacy QTLs for grain Cd and Zn/Cd ratio mapped using field-grown plants (Liu et al. 2020; Zhang et al. 2014, 2011), and with an a priori candidate gene, *OsHMA2*, which encodes a member of the P_1B_-ATPase transporter family. The POCRE SNPs are located 4.1 kb upstream of transcript1 (SNP-chr6:29,475,763) and within the first exon of transcript2 (SNP chr6:29,479,354) of *OsHMA2,* where the latter is predicted to cause a synonymous substitution (Suppl. Table S-1). Regional GWA analysis in the *tropical japonica* subpopulation using the high-density imputed SNP dataset supports a plateau of significant SNPs in LD surrounding *OsHMA2* (Suppl. Figure S-3b). Haplotype analysis identified five haplotypes across the region, with four represented in the *tropical japonica* subpopulation (Suppl. Figure S-3c). Within *tropical japonica,* Hap4 had significantly lower Cd_shoot than other haplotype groups (ANOVA *p* value < 0.001) (Suppl. Figure S-3d) and SNPs defining the GWAS peak were in LD across the region. As shown in Supplementary Figure S-3e, the T/T allele at SNP chr6:29,480,530, which maps within the 5’UTR of HMA2, distinguishes Hap4 from the other haplotype groups and is associated with lower Cd-shoot concentration.

More surprisingly, when GWA was run individually on each subpopulation, an additional peak associated with Cd_shoot was identified on chromosome 7 in *aus* (Peak#87 in Suppl. Table S-1). This peak was detectable as a sub-threshold signal in the INDICA clade (though also supported by a POCRE SNP in that analysis) but rose to significance in the *aus* subpopulation (*p* value < 5.48E-8) (Suppl. Figure S-4a and S-4b). Using the high-density SNP dataset for chromosome-specific GWA in *aus,* we observed an increase in the number of significant SNPs associated with Cd_shoot in the target region (Suppl. Figure S-4c). Haplotype analysis revealed 10 distinct haplotypes in the RDP1 panel as a whole across the 31.3 kb region (data not shown), suggesting that this is a region of unusually high diversity in the rice genome. Five of the ten haplotypes were found among INDICA lines: Hap1 was private to *indica,* Hap2 was shared between *indica* and *aus,* and Hap3, Hap4, and Hap5 were private to *aus* (Suppl. Figure S-4d). Despite the very small sample size, a highly significant difference in Cd_shoot was observed in lines carrying *aus*-specific Hap5 compared to any of the other haplotype groups, with Hap5 conferring higher Cd_shoot (Suppl. Figure S-4e). The region contains no *a priori* candidate genes. The three most significant SNPs fall within LOC_Os07g36060, which is annotated as an SMP-30 Gluconolactonase LRE-like region-containing protein. Further research is needed to validate these results in a larger number of lines, but our findings identify a putative haplotype associated with high Cd_shoot that is unique to the *aus* subpopulation and narrow the search space for a causal gene to a ~ 100 kb region.

### Shoot molybdenum

**Fig. 6:**
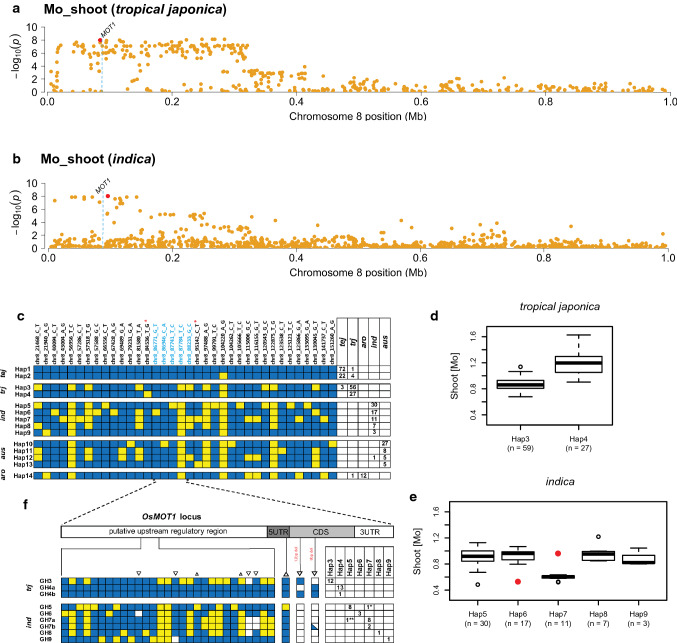
Haplotype analysis of a region on rice chromosome 8 associated with Mo_shoot containing the *Os-MOT1;1* locus.** a** Zoom-in of chromosome 8 (0.02–1.0 Mb) showing GWA peak in *tropical japonica* and **b** in *indica*; dotted blue lines indicate position of *MOT1* gene; gold dots represent SNP p values from GWAS using the unimputed HDRA SNP dataset; red dots represent MS-SNPs in GWA for each subpopulation, respectively. **c** Haplotype analysis of 130 kilobase region extending across *Os-MOT1;1* region; blue boxes = reference (Nipponbare) alleles; yellow boxes = alternate alleles; SNPs labeled in blue map within the *Os-MOT1;1* gene model; red asterisks indicate MS-SNPs in GWA for *tropical japonica* and *indica,* respectively. Boxes to right indicate number of lines carrying each haplotype within each subpopulation. **d** Quantile boxplots show phenotypic distribution of Mo_shoot content in haplotype groups found in *tropical japonica* and **e** in *indica;* red dots indicate phenotypic outliers corresponding to rare variants that carry a different gene-based haplotype at *Os-MOT1;1.* **f** Gene-based haplotype analysis of *Os-MOT1;1*; white cells = deletions relative to reference; triangles above rows = 1-bp insertions; inverted triangles above rows = deletions of various sizes; CDS deletion-sizes indicated in red text; blue triangle = 3-bp deletion in same locus carrying 9-bp deletion for GH7b; boxes to right indicate number of lines carrying each gene-based haplotype (GH); asterisks indicate phenotypic outliers (corresponding to red dots in (e)) with gene-based haplotypes that differ from their regional haplotypes

Our GWA analysis identified a Tier 1 peak for Mo_shoot on rice chromosome 8 (peak #48 in Suppl. Table S-1) in the genomic region of the *Os-MOT1* gene (LOC_Os08g0112). This was the only case where a significant association was observed in ALL (*p* value < 10E-17), as well as in both the INDICA and the JAPONICA clades. Within JAPONICA, the region was significant in the *tropical* but not the *temperate japonica* subpopulation, and in INDICA, it was significant in *indica* but not in the *aus* subpopulation (Fig. 6a and b; Manhattan Plots: https://www.zstats.org/rice/index.html). The peak was supported by three SNPs detected by the POCRE multi-marker analysis, the presence of a biparental QTL from the IR64 x Azucena RIL analysis, a legacy QTL reported in Yang et al. (2018), and the rice ortholog of *AtMOT1;1* (*Os-MOT1;1*) (Suppl. Table S-1).

We next examined variation in the region around *Os-MOT1* to understand the subpopulation context of the GWA peak. Based on imputed genotypes, we constructed haplotypes encompassing a 130 kb region under the GWA peak (Fig. 6c). A total of 14 haplotypes were identified and grouped as *temperate japonica* (Hap1 & Hap2), *tropical japonica* (Hap3 & Hap4), *indica* (Hap5–Hap9), *aus* (Hap10–Hap13), and *aromatic* (Hap14), according to the subpopulation in which each was detected at highest frequency. In the *tropical japonica* and *indica* subpopulations where significant GWA peaks were observed, we next compared the mean Mo_shoot phenotypes among haplotype groups within each subpopulation (Fig. 6d and 6e). Two haplotypes were observed in *tropical japonica,* and lines carrying Hap4 had significantly higher Mo_shoot (Welch t-statistic: 4.11E-10) than lines carrying Hap3 (Fig. 6d). Of the five haplotypes observed in *indica,* lines carrying Hap7 had significantly lower Mo_shoot values (p value from bootstrapped ANOVA: 1.04E-10) than lines carrying any of the other haplotypes (Fig. 6e).

To further characterize genetic variation at the *Os-MOT1;1* locus and identify potential functional polymorphism(s) driving the observed phenotypic variation, we performed Sanger sequencing across the candidate *Os-MOT1;1* locus in 26 *tropical japonica* and 25 *indica* varieties selected to represent the different haplotypes shown in Fig. 6 (see Suppl. Table S-5). We sequenced the upstream regulatory region (1 kb), the 3’UTR (1 kb) as well as the coding region (CDS) of *Os-MOT1;1*. The sequencing detected 29 polymorphisms in the upstream regulatory region, a 10-bp insertion in the 5’UTR, two deletions in the CDS, and no variants in the 3’UTR. These variants were used to construct gene-based haplotypes (GH) for the *tropical japonica* and *indica* subpopulations (Fig. 6f).

In the *tropical japonica* subpopulation, 12 SNPs and one deletion in the putative upstream regulatory region, a 9-bp and a 12-bp deletion in the CDS differentiated GH3 and GH4 (Fig. 6f). The 9-bp deletion was common to all 12 lines carrying Hap3; this deletion is predicted to cause an in-frame mutation in *Os-MOT1*, removing three of eight consecutive Glutamine residues in the protein product (at positions 215–218). The 12-bp deletion was private to GH4. Thirteen lines carrying the deletion were classified as GH4a; one Hap4 line did not carry the deletion but was otherwise identical to the others and was classified as GH4b (Fig. 6f**).** There was no phenotypic difference between lines carrying GH4a and GH4b, so we next compared the phenotypic means of the 14 *tropical japonica* lines carrying GH4 with the 12 lines carrying GH3 and discovered that lines carrying GH4 had significantly higher Mo_shoot values than lines carrying GH3 (Welch *t*-statistic < 0.0001), consistent with the pattern of phenotypic variance observed with Hap4 and Hap3. To further test this hypothesis, we used the haplotypes from the 26 sequenced *tropical japonica* lines to infer the *OsMOT1* haplotypes carried by an additional 62 lines for which we had high-density HDRA SNP data and phenotypic information. We then examined the difference between lines carrying GH4 and those carrying GH3, and again observed that GH4-containing lines had higher Mo_shoot than GH3 containing lines (data not shown).

In the *indica* subpopulation, 23 SNPs and 4 indel variants in the upstream regulatory region, one SNP in the 5’ UTR, and one 3-bp indel in the CDS differentiated the haplotypes (Fig. 6f). All sequenced *indica* lines carried deletions in the CDS corresponding to the locations of the 12-bp and the 9-bp deletions mentioned above. Variation within GH7 differentiated two lines: one carrying a 3 bp (rather than a 9 bp) deletion in the CDS and one line carrying no deletion; those with the 9-bp deletion were labeled as GH7a and those carrying the 3-bp deletion at that location were designated as GH7b. The difference in the size of the deletion does not appear to be associated with a meaningful phenotypic difference among these lines. While the functional polymorphisms cannot be determined based solely on natural variation at this locus within this germplasm panel, it is clear from our results that variation in *indica* is almost entirely associated with the upstream regulatory region, which is consistent with previous reports of expression level polymorphism driving Mo_shoot concentration in this subpopulation (Yang et al. 2018). However, the association we report in the JAPONICA clade is novel compared to previous reports of this locus and illustrates that functional variation in the CDS region cannot be ruled out as causal in the *tropical japonica* subpopulation (see discussion below).

## Discussion

### A multi-pronged approach to genetic signal detection

Understanding the genetic control of mineral nutrient acquisition in rice is critical as breeders, geneticists, physiologists, and other plant biologists seek to improve the climate resilience, nutritional quality, and productivity under changing crop management regimes. Schroeder et al. (2013) proposed that membrane transporters are key targets for improving the efficiency with which plants take up and use water and nutrients, with the ultimate goal of improving crops for sustainable food production. In fact, along with transcription factors, membrane transporters have emerged as one of the major classes of genes predominant as determinants of abiotic stress tolerance (Mickelbart et al. 2015). Root tissue is particularly important to understand in this context as it sits at the interface between the physical and biological environments. Despite the critical importance of roots in this system, no systematic interrogation of the genetic mechanisms of all biologically relevant ions has ever been done in root tissue in rice (Campbell et al. 2017; Lin et al. 2004; Patishtan et al. 2018; Wang et al. 2012; Zheng et al. 2015).

Our rationale for using a controlled environment to do such an investigation was threefold: first, to control signal to noise ratios to better resolve small phenotypic differences, secondly to enable analysis of both root and shoot tissue from the same plant, and third to produce a set of testable hypotheses across a broad range of phenotypes and germplasm that could be interrogated in more targeted ways using field-based approaches and/or perturbations of specific ion concentrations in the future. This approach paid significant dividends in the detection of novel genetic signal associated with root ion concentration that would have been untenable or impossible to achieve using field-grown plants.

The almost complete lack of overlap between root and shoot genetic associations implies that surveys limited to above ground tissue are not able to fully characterize the suite of complex genetics that characterize the rice ionome. The rare instances of overlapping QTL for the same element in both root and shoot tissue detected in the IR64xAzucena biparental mapping analysis are likely driven by low mapping resolution and may be due to different genes contributing to the tissue-specific variation associated within these large regions. The comparatively high mapping resolution of genome-wide association in the RDP1 permits the investigation of this question because linkage blocks are substantially smaller than in the RIL population (i.e., compare Fig. 2 and Suppl. Figure S-2). Additionally, because the IR64xAzucena population is derived from an INDICA by JAPONICA cross, it brings together clade-specific (or subpopulation specific) variation not commonly observed in historical collections, opening the door to unexpected transgressive outcomes. In fact, two instances were observed where GWA peaks for one tissue overlapped with biparental QTL for the same element (Mo and Mn) in the opposite tissue. The fact that genetic signal in the same genomic regions is detected from opposing tissues in these two sets of genetic materials suggests that the variation at these loci may have evolved independently in the INDICA or JAPONICA clades. If so, the use of wide crosses is likely to expose phenotypic variation for ion concentrations that would not be observed in a GWA panel where the clades and subpopulations retain their identity, as is the case here.

Given the sparsity issues created by the “large *p* small *n*” challenge associated with GWA studies, approaches that evaluate the significance of one SNP at a time are prone to Type II error. To address this challenge, a multi-marker method (POCRE) was employed that can simultaneously evaluate all SNPs for their association with the phenotype, and then combine with other approaches to obtain statistical significance. Taking advantage of the supervised dimension reduction approach and Bayesian inference (Zhang et al. 2005), POCRE efficiently selects the associated SNPs from a much larger number of candidates in the face of potential LD between SNPs (Zhang et al. 2009a, b). Briefly, POCRE first constructs orthogonal components that are most closely related to the phenotype, and a new penalization framework using empirical Bayes thresholding can detect the important SNPs in each component (Johnstone and Silverman 2005, 2004; Zhang et al. 2010). Such construction continues until there is no correlation between the component and the phenotype. As a data-driven approach, POCRE is computationally efficient due to the sequential construction of orthogonal components. While POCRE is developed as a screening strategy for general high dimensional, low sample size data, it has been successfully employed in human GWA data analysis (Lin et al. 2009; Zhang et al. 2009a, b), genomic selection in pine and maize (Pungpapong et al. 2012), and eQTL analysis in Arabidopsis (Wang et al. 2020). Compared to other methods for multi-SNP-based analysis, POCRE is both computationally efficient and exhibits a low FDR (Pungpapong et al. 2012; Zhang et al. 2009a, b).

In this study, we demonstrated that by combining high-quality genetic and genomic resources, controlled environment phenotyping, Bayesian modeling, and multi-marker analysis it’s possible to produce high-quality rice association data that permits the formulation of specific testable hypotheses. The use of multiple empirical strategies in combination is relatively rare (Zheng et al. 2015), but proved useful in this study for lending more confidence to GWA peaks than can be typically attributed to a single-marker analysis. The use of subpopulation-specific GWA and haplotype analysis, while underpowered, were useful for interpreting the genetic signal detected in ALL or in the clade-specific analyses, as demonstrated by the strong signal for S_shoot, Mn_root, and Mo_shoot analyses where we detected highly significant associations in ALL that were ultimately explained by subpopulation or clade-specific variation. Furthermore, when subpopulation-specific alleles occur at low frequencies in ALL, they may go undetected unless GWA is performed at the level of the clade or subpopulation, such as the novel *aus* peak for Cd_shoot undetected in ALL but of high significance in the *aus* subpopulation, underscoring the importance of these partitioned analyses in *O. sativa*. This strategy allowed us to quickly focus in on genomic regions for four elements that exhibited the strongest evidence for causal association.

### Shoot sulfur

While the physiological interplay between members of the sulfate transport pathways in rice is not currently well understood (Mitani-Ueno et al. 2018), there is evidence from studies in *Arabidopsis* indicating that *SULTR1*-type transporters are essential for uptake of sulfur from the soil (Shibagaki et al. 2002; Takahashi et al. 2000) while *SULTR2*-type transporters may play a more critical role for translocation of sulfur from the root to above ground tissues (Bell 2008; Kataoka et al. 2004; Maruyama-Nakashita et al. 2015; Rausch and Wachter 2005; Takahashi et al. 2000). This is consistent with the results of our study as the MS-SNP detected using the imputed regional GWA analysis for the S_shoot phenotype was found to be 24 bp downstream of the *SULTR2;1* candidate gene. (Fig. 4a; Manhattan Plot for S_shoot at https://www.zstats.org/rice/index.html). This outcome highlights the value of using higher-density imputed genotype datasets to improve the interrogation of potentially functional polymorphisms.

The signal for this peak was among the most significant single-marker p values observed in our analysis (Suppl. Table S-1; Manhattan Plot for S_shoot at https://www.zstats.org/rice/index.html), however the peak is only present when considering the entire panel (ALL), while clade and subpopulation-specific analyses did not detect it. Haplotype analysis of this region proved useful in understanding this pattern where we discovered the primary determinant of S_shoot was Hap3 which is private to the *aromatic* group (Fig. 4b). The aromatic group has too few lines in our panel for independent subpopulation analysis and is not considered part of either the INDICA or JAPONICA clades. The phenotypic effect for Hap3 was further implicated by the single *indica* line in our analysis that possessed an introgression containing Hap3 at this region and was also a phenotypic outlier in that clade. Interestingly, five out of the seven lines carrying Hap3 (all aromatic) also exhibited as heterozygotes for the MS-SNP and have an intermediate S_shoot phenotype (Fig. 4e). This supports the hypothesis that alleles at this locus exhibit a dosage effect resulting in a co-dominant gene action associated with the phenotypic effects on S_shoot. A thorough investigation among sequenced lines to understand the extent of natural genetic variation at this locus and its effect on the concentration of sulfur in above ground tissue would be warranted to better understand its phenotypic effects.

### Root manganese

Manganese (Mn) is a required micronutrient for all organisms, and a transition metal that acts as a protein cofactor catalyst in electron transfer reactions. Mn is a constituent of essential metalloenzymes, including the antioxidant defense enzyme superoxide dismutase, and is part of the complex that catalyzes water oxidation in photosystem II (Marschner 2012). Like all essential transition metals, Mn in excess can also be toxic to plants. High concentrations of Mn can cause chlorosis, brown speckles on mature leaves, and necrosis, which result in reduced crop yield (Marschner 2012). Certain rice varieties are able to tolerate the accumulation of high Mn concentrations in their tissues (Foy 1992).

The strongest peak in our analysis of Mn_root was peak #17, which was detected in ALL and persisted only when the *tropical japonica* subpopulation was analyzed independently, implying that the peak in ALL is driven by variation in *tropical japonica.* (Manhattan Plot for S_shoot at https://www.zstats.org/rice/index.html). Among the *a priori* candidate genes that co-localized with this GWA peak, *OsNramp5* stands out as the most likely candidate for functional genetic variation. The rice gene *OsNramp5* has been shown to be responsible for Mn uptake in roots, and the knockout mutant shows reduced Mn accumulation, reduced growth, and a significant reduction in yield component traits (Ishimaru et al. 2012; Sasaki et al. 2012; Yang et al. 2019). The rice *OsNramp5* gene is also implicated in Cadmium uptake (Ishimaru et al. 2012; Peris-Peris et al. 2017; Sasaki et al. 2012) although in our study we did not detect a significant association with Cd content in this genomic region.

Our analysis was unable to resolve the genetic signal to differentiate the causal significance of *OsNRAMP5* vs *OsNRAMP1,* as all variants within these genes in our dataset were in LD among our lines*.* While homologs of *OsNRAMP1* have been shown to transport Manganese as well as other divalent cations in Arabidopsis (Cailliatte et al. 2010) and peanut (Wang et al. 2019), the ability of *OsNRAMP1* to transport Manganese in rice is questionable based on the inability of the Nipponbare allele (*temperate japonica*) to restore intra-cellular Mn concentrations when cloned into yeast mutants deficient in Mn transport capacity (Takahashi et al. 2011). Our analysis revealed that the Nipponbare haplotype is the most common haplotype among *temperate japonica* lines, and that an array of naturally occurring alleles across subpopulations of rice (including a haplotype unique to the *aromatic* subgroup and a rare haplotype only found in a minority of *tropical japonica* lines) have up to 40% higher root Mn concentrations compared to lines possessing the Nipponbare haplotype. Given the extent of natural variation at this locus and the fact that the MS-SNPs for this trait flank *OsNRAMP1* in our analysis (Fig. 5a), we suggest that a more thorough investigation into the effects of natural variation at *OsNRAMP1* on root Mn concentration is needed before its role in Mn uptake and transport in rice can be fully ruled out.

Interestingly, there was also strong genetic signal from peak #23 (5.5 Mb downstream of peak #17) that persisted in the *tropical japonica*-specific GWA analysis. Reminiscent of the situation with S_shoot, we identified a rare subpopulation-specific haplotype (Hap3; Fig. 5e) with a large phenotypic effect on Mn_root concentration. However, the low number of lines possessing Hap3 and the extent of LD across this region did not allow us to fully dissect the genetic signal to differentiate peak #23 from peak #17. To test the hypothesis that the signal for peak #23 might be due to extended LD from peak #17, we identified two rare lines carrying a recombination breakpoint between peak #17 and peak #23 (1 + 3 and 3 + 1, respectively, in Fig. 5e). When associated with phenotypic variation for Mn_root, both recombinant haplotypes had an intermediate phenotype, suggesting that alternate alleles across peaks #17 and #23 contribute equally, and in an additive fashion, to the high Mn_root phenotype associated with Hap3-containing lines. The significance of this genomic region for Mn-shoot/grain concentration has been previously reported by Yang et al. (2018). Using GWA and haplotype analysis from the imputed genotype data allowed us to resolve this peak further and to provide stronger evidence for the role of these two *OsNRAMP* genes in Mn transport in rice, as well as the additive value of the larger genomic region. The fact that Yang et al. (2018) identified this region using genetic signal from above ground tissue, while our analysis detects significance in root tissue posits that this region could play a significant role in Mn translocation as well as uptake from the soil solution. Furthermore, in an inbreeding species like rice, where large LD blocks tend to be inherited together more frequently than in outcrossing species, maintaining tandem arrays of genes whose collective, additive effects contribute to large phenotypic impacts may provide an evolutionary advantage for adaptation to complex environments. Such arrays would be of particular interest to breeders aiming to fortify new varieties against micronutrient deficiencies in marginal environments (i.e., acid soils) or to biofortify new varieties with enhanced mineral nutrition.

### Shoot cadmium

Natural variation occurs in both the uptake and the distribution of Cd in crop species, including rice. While *OsHMA3* (chromosome 7) is among the most widely studied genes in rice due to the strong role it plays in cadmium toxicity tolerance and its ability to sequester cadmium into root vacuoles (Ueno et al. 2010), our analysis detected a GWA peak for Cd-shoot concentration on chromosome 6, closely associated with *OsHMA2* (Suppl. Figure S-3). The effect of this chromosomal region on Cd-related phenotypes is well supported by previous functional genomics studies in rice and by GWA signal from grain tissue among a limited number of USDA lines reported by Liu et al. (2020). Importantly, knockouts of this transporter have also been shown to substantially reduce cadmium concentration in above ground tissues, including shoots and grain (Adil et al. 2020; Satoh-Nagasawa et al. 2012; Takahashi et al. 2012; Yamaji et al. 2013). Interestingly, Liu et al. (2019) conducted a GWA analysis using a subset of the same lines and SNPs selected for this study and did not detect association between the *OsHMA2* region and grain Cd concentration despite using the same genetic and genomic resources and measuring a similar broad-sense heritability for Cd concentration (0.35). The discrepancy is likely explained by differences in phenotyping (grain harvested from heavily contaminated farmland vs shoot tissue harvested from hydroponic growth conditions with sub-toxic Cd levels). This key phenotyping difference leads to the elucidation of different genotype–phenotype relationships and highlights the utility of controlled/managed environments for positing hypotheses that can be later tested using functional genomics approaches and more targeted field experiments.

Notably, our analysis also identified an unexpected, novel association with Cd_shoot concentration on chromosome 7 in the *aus* subpopulation driven by a rare haplotype (Hap5; Suppl. Figure S-4d). The large phenotypic effect coupled with the rarity of this haplotype provides another example of the value of subpopulation-specific GWA analysis in rice. Larger panels that disregard subpopulation structure or limit resolution to only the INDICA and JAPONICA clades would have eliminated lines carrying this variant due to low minor allele frequency. Despite reduced power to detect *aus-*specific associations in our panel (as only 52 *aus* lines remain under consideration when such filters are applied), combining GWA with imputed haplotypes allowed us to identify a rare *aus* haplotype and pose hypotheses about potentially functional variation. While such small sample sizes do not preclude the possibility of artifactual associations, the case is consistent with previously documented instances in rice where rare alleles detected in subpopulation-specific GWA analyses underly previously undescribed functional variation (Famoso et al. 2011; Li et al. 2014).

### Shoot molybdenum

The few known molybdate-specific transporters in plants belong to the *Molybdate Transporter 1* (*MOT1*) family, first identified in the green alga *Chlamydomonas reinhardtii* (Tejada-Jimenez et al. 2007) and in *Arabidopsis thaliana* (Baxter et al. 2008; Tomatsu et al. 2007). More recently, *MOT1* homologs were identified in *Lotus japonicas* (Duan et al. 2017; Gao et al. 2016) and *Medicago* (Tejada-Jimenez et al. 2017). In rice, recent association mapping studies focusing on ionomic variation in vegetative tissues and rice grain collected from field-grown plants (Norton et al. 2014; Yang et al. 2018) both found significant associations for Mo in the region containing the *MOT1* homolog on rice chromosome 8. Yang et al. (2018) further demonstrated that variation in molybdenum accumulation in rice leaf and grain tissue is caused by variation in expression levels of *Os-MOT1;1*, similar to findings in *Arabidopsis*.

Our GWA analysis identified a strong genetic signal for Mo_shoot concentration in this region as well. Similar to the analysis done by Yang et al. (2018), this peak (peak #48 in Suppl. Table S-1) was the strongest and clearest peak in our analysis. Notably, we discovered variation at this locus that correlates with phenotypic differences in Mo_shoot in both the INDICA and JAPONICA clades, and specifically in the *indica* and *tropical japonica* subpopulations. The gene haplotypes constructed based on targeted sequencing of the *Os-MOT1;1* locus in the RDP1 panel proved to be better predictors of the Mo_shoot phenotype than the original haplotypes identified using imputed genotype data due to recombination in the regions flanking the causal gene. For example, a rice line from China (Ai-Chiao-Hong; NSFTV3) that belongs to a low Mo_shoot haplotype (Hap7 in Fig. 6f, indicated by an asterisk) appears to carry a high Mo allele at the *Os-MOT1;1* locus (GH5, one of the high Mo_shoot haplotypes commonly associated with high Mo haplotype, Hap5). Similarly, another rice line from China (Aijiaonante; NSFTV234) that possesses Hap5 has an uncharacteristically low Mo_shoot concentration (outlier in the boxplot in Fig. 6e; indicated by two asterisks in Fig. 6f), also carries the gene haplotype GH7a at the *Os-MOT1;1* locus, associated with high Mo_shoot values. These results demonstrate that the sequence-based gene haplotypes at the *Os-MOT1;1* locus are better predictors of phenotypic outcomes than the regional haplotypes, consistent with the nature of recombination around a causal gene and supporting the conclusion that variation in *Os-MOT1;1* underlies the phenotype.

Using the germplasm panel reported by Chen et al. (2014), Yang et al. (2018) detected a strong genetic association between straw and grain molybdenum content among *indica* cultivars but found no association among either *temperate* or *tropical japonica* lines. Our results differ from Yang et al. (2018) as we do observe significant and strong association between the *Os-MOT1;1* region and Mo_shoot among *tropical japonica* cultivars (Fig. 6a). In fact, one of the two *tropical japonica* haplotypes we report (Hap3) bears notable resemblance to the *indica* haplotype Hap8 (Fig. 6c), suggesting that the driver of low shoot molybdenum content in *tropical japonica* is the result of an introgression, most likely originating from a Hap8-carrying *indica* line. In contrast, within *indica,* variation from Hap7 (rather than Hap8) drives lower Mo content. This underscores the impact of genetic background, such that the same haplotype may have a different phenotypic impact in the *indica* versus the *tropical japonica* gene pool, and is consistent with the fact that *indica* lines have overall lower shoot Mo content.

Upon sequencing a subset of lines from their analysis, Yang et al. (2018) found significant variation in the promoter region upstream of *Os-MOT1;1*, but no variants within the CDS of the gene among the *indica* lines they analyzed. Using reverse genetic approaches, they convincingly demonstrated that expression variants within *indica* were the primary drivers of shoot Mo content in their germplasm. Our analysis supports this assertion as we also discovered significant variation in the upstream regulatory region of *Os-MOT1;1* among both *indica* and *tropical japonica* lines (Fig. 6c and f), suggesting that differences in expression levels of this gene likely impact shoot molybdenum content. Importantly, we also discovered several polymorphic sites within the coding region of the gene, including a 9-bp deletion in the CDS which is polymorphic among *indica* lines (GH7). Additionally, we identified a second position within the CDS (a 12-bp deletion), present only in *tropical japonica* lines (GH4a; Fig. 6f). This suggests that variation within the *Os-MOT1;1* CDS region exists and it would be worth investigating whether it contributes to variation for shoot Mo content within a larger sample of germplasm. Further studies examining *Os-MOT1;1* coding region variation as well as expression level differences in diverse rice varieties are necessary to confirm how this gene contributes to phenotypic variation within both the *tropical japonica* and *indica* subpopulations.

## Conclusion

Understanding the genetic architecture of the ionome opens the door to manipulating complex but poorly interrogated traits such as nutrient acquisition. Considering variation across tissues and ancestral subpopulations allowed us to discover interesting and non-intuitive relationships that illuminate the complex network of correlated phenotypes that comprises the broader ionome. The data, genetic resources, and analytical results presented in this study establish a series of baseline datasets using a repeatable and controlled phenotyping platform and provide a vital community resource. Using this analysis as a starting point, clear hypotheses can be developed and tested using perturbations of both environmental and/or genetic variation and its impact on phenotypic outcomes. For example, it’s unclear how elemental deficiencies and toxicities may impact the function and importance of dual-purpose transporters with competing affinities when considered in combination with the varied and deeply differentiated genetic backgrounds present in rice. Our work highlights intersections between multiple lines of empirical evidence and offers clear justification for the investment of additional resources to further explore the functional significance of key components of genetic variation to understand its impact on the ionome and ultimately, how the genotype mediates plant interactions with the biophysical world.

Most crop improvement programs struggle to strike a balance between the benefit of introducing novel allelic variation for traits of interest and the unintended cost that novel variation can have on non-target traits in elite gene pools. This work provides a valuable road map to facilitate targeted introduction of specific alleles into elite genetic backgrounds using either marker-assisted backcrossing or genome editing. The use of editing makes it possible to test genetic hypotheses without the genome-wide disruption that inevitably occurs when novel alleles are introduced through backcrossing. Editing targets identified herein also provide useful points of entry to explore and better understand the kind of transgressive interactions that frequently occur when rare alleles originating in one subpopulation of rice are introduced into a different subpopulation or genetic background. In either case, the resources developed and described in this study provide a valuable foundation for exploring, understanding, and mobilizing natural variation associated with the root and shoot ionomes of domesticated Asian rice.

## Supplementary Information

Below is the link to the electronic supplementary material.

Supplementary file1 (PDF 3918 KB)

Supplementary file2 (XLSX 45 KB)

Supplementary file3 (XLSX 14 KB)

Supplementary file4 (XLSX 22 KB)

Supplementary file5 (XLSX 59 KB)

Supplementary file6 (XLSX 180 KB)

Supplementary file7 (XLSX 17 KB)

## Data Availability

Data are available at https://www.zstats.org/rice/index.html.
