# Supplementary material for: Genetic architecture of root and shoot ionomes in rice (Oryza sativa L.)
Source: Theor Appl Genet. 2021 May 20;134(8):2613–37. doi: 10.1007/s00122-021-03848-5 (PMC8277617; doi:10.1007/s00122-021-03848-5)
Supplement: Supplementary file 1 — Supplementary file1 (PDF 3918 KB) [file 122_2021_3848_MOESM1_ESM.pdf]

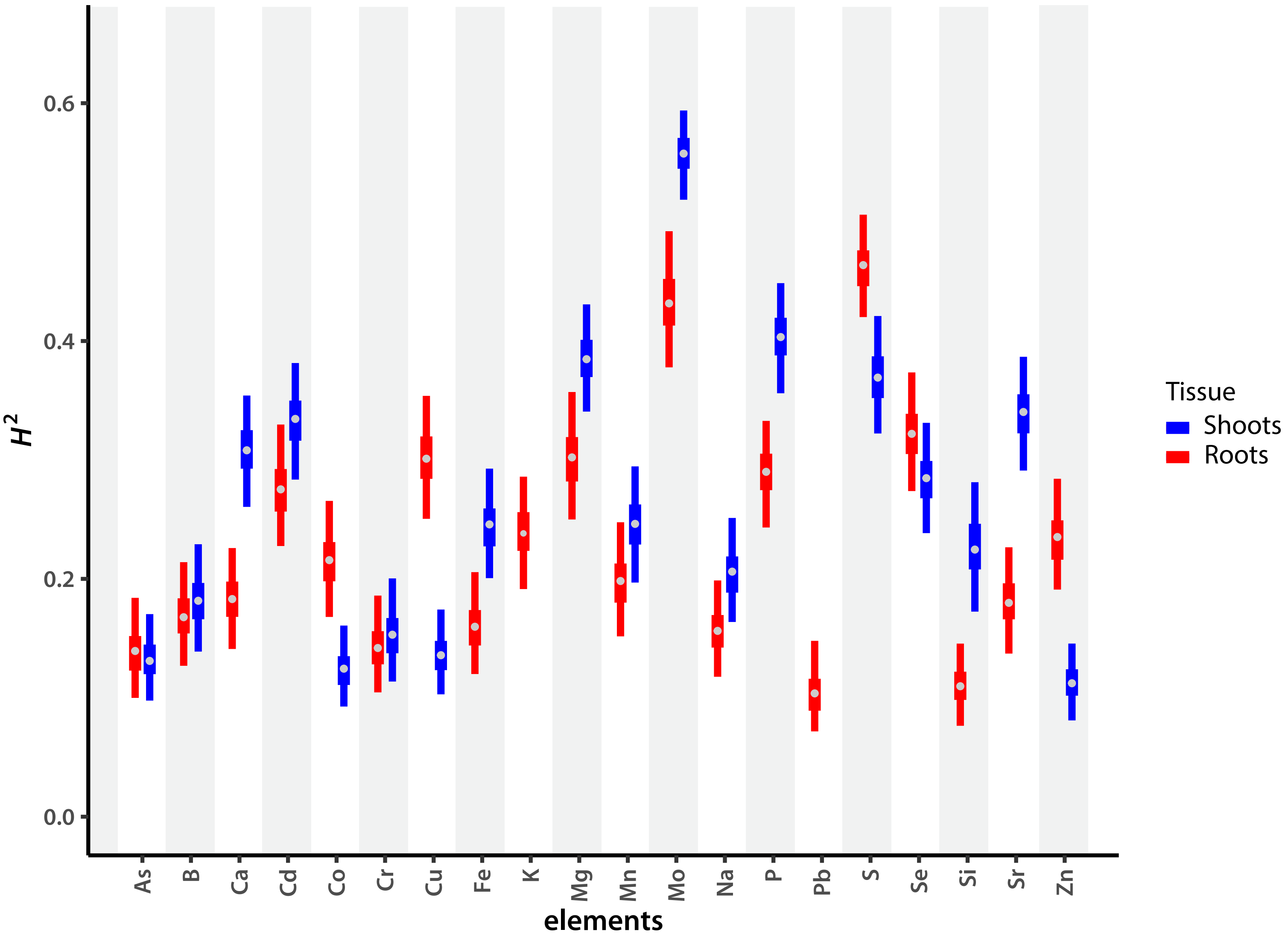

Supplementary Figure S-1: Plot of broad-sense heritabilities measured for each of the 20 ionomic phenotypes in roots and shoots. Heritability estimates represent Bayesian approximations of broad-sense heritability (H^2 = Var(among lines)/[Var(among lines) + Var(among replicates)]) of shoot (shown in blue) and root (shown in red) phenotypes; grey dots represent the mean of the posterior distribution; whiskers represent the variance of the same.

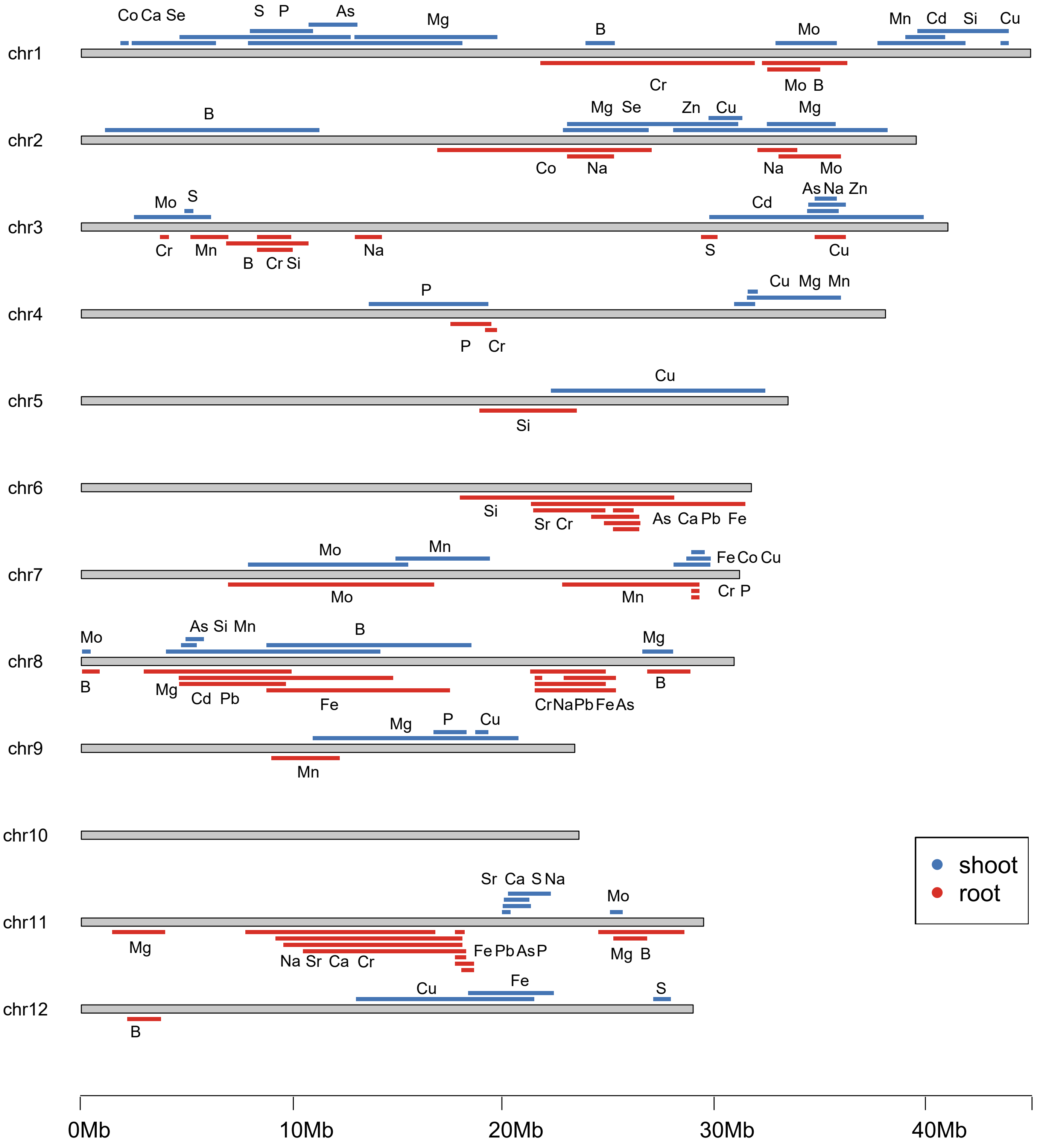

Figure S-2: Graphical representation of QTL intervals detected in the biparental (IR64 x Azucena) QTL analysis. Red bars = root QTLs; blue bars = shoot QTLs; elements are abbreviated as in Table 1; labels for overlapping QTL are ordered according to the start of the QTL interval along the chromosome, as in Supplementary Table S-1.

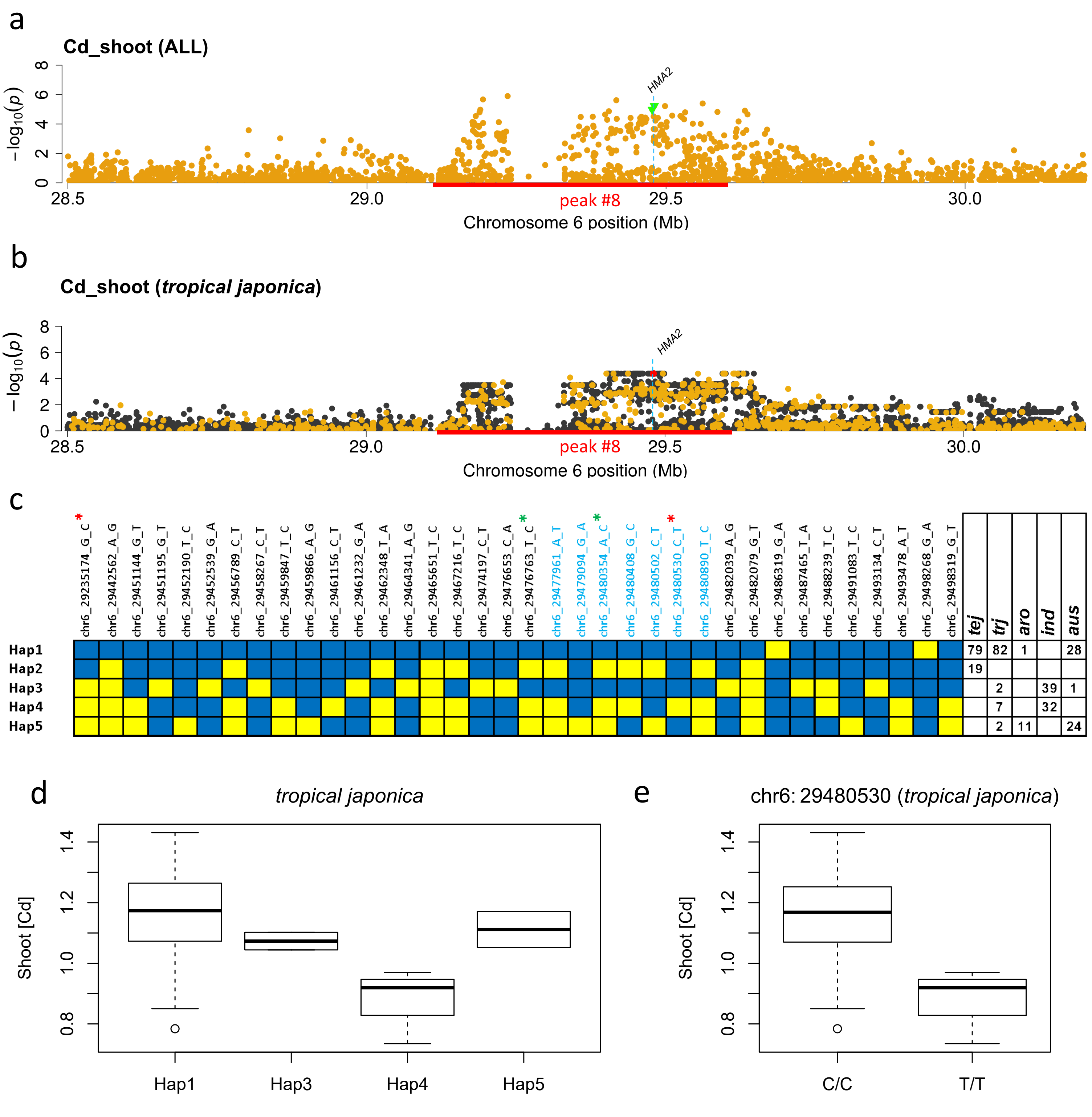

Supplementary Figure S-3: A region associated with Cd_shoot co-localizes with *OsHMA2* on rice chromosome 6. (a) Zoom-in of chromosome 6 (28.5 – 30.2 Mb) showing GWA peaks in ALL; dotted blue line indicates the position of *OsHMA2*; gold dots represent SNP p-values from GWAS using the unimputed HDRA SNP data set; horizontal red bar = region of GWA peak (#8); inverted green triangles = SNPs identified by POCRE. **(b)** Zoom-in of same region for *tropical japonica*; gold dots indicate SNPs from unimputed SNP dataset; black dots indicate SNPs from imputed SNP dataset; red dot = MS-SNP. **(c)** Haplotype analysis of 263 Kb region containing *HMA2*; blue boxes = reference (Nipponbare) alleles; yellow boxes = alternate alleles; SNPs labeled in blue map within *HMA2*; red asterisks = MS-SNPs in ALL (SNP-6:29235174) and in *tropical japonica* (SNP-6:29480530); green asterisks = POCRE SNPs; boxes to right indicate number of lines carrying each haplotype. **(d)** Quantile boxplots show phenotypic distribution of Cd_shoot content in haplotypes found in tropical *japonica*. **(e)** Quantile boxplots show phenotypic distribution of the two genotypic classes detected by MS-SNP (SNP-6:29480530).

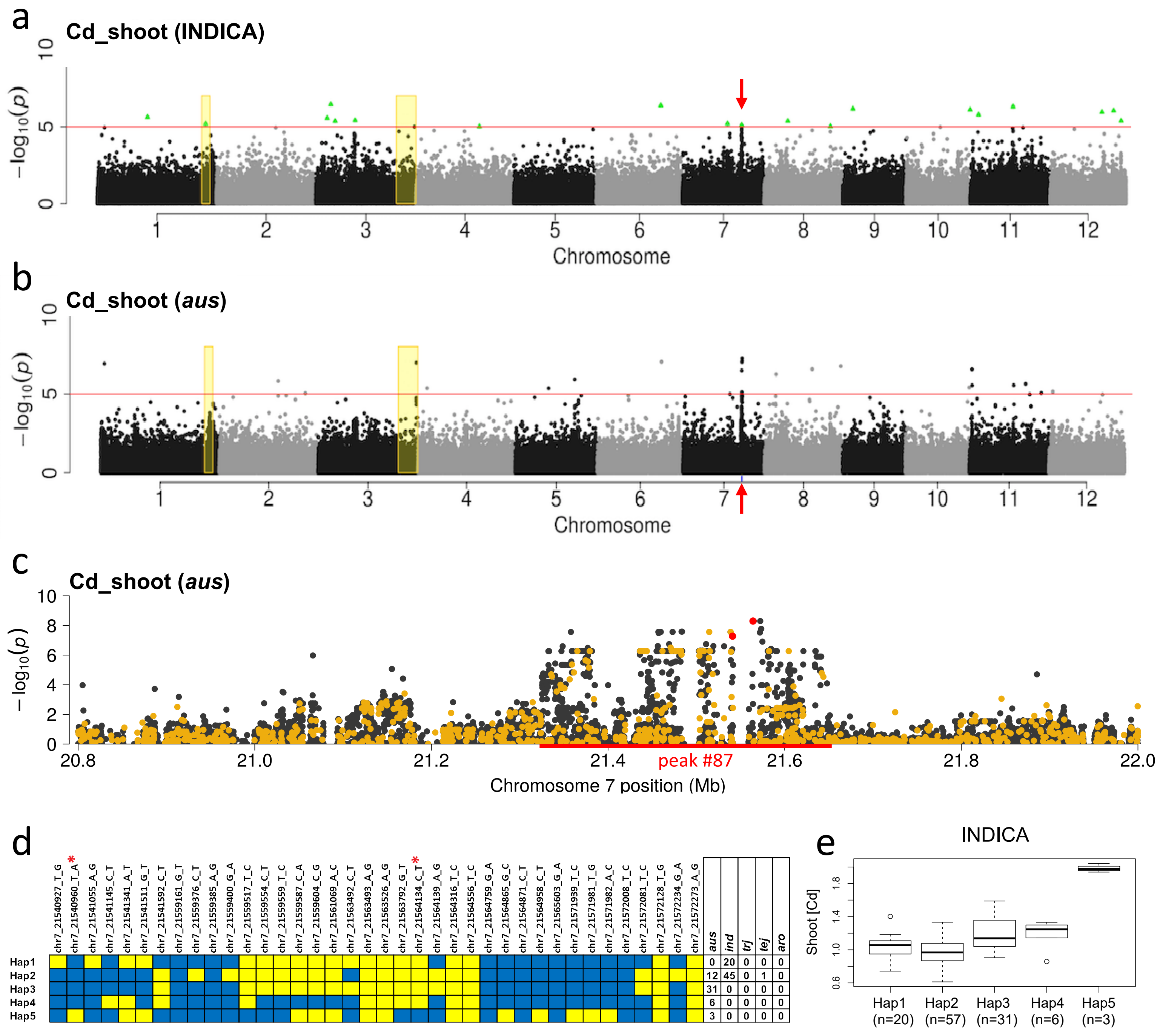

Supplementary Figure S-4: A region associated with Cd_shoot in the *aus* subpopulation on rice chromosome 7. (a) GWA for Cd_shoot concentration using the HDRA SNP dataset for the INDICA clade; vertical red arrow = peak of interest; yellow transparent rectangles = legacy QTL; red horizonal line = significance threshold; green triangles = POCRE SNPs; **(b)** GWA for Cd_shoot concentration using the HDRA SNP dataset for the *aus* subpopulation; vertical red arrow = peak of interest; yellow transparent rectangles = legacy QTL; red horizonal line = significance threshold; green triangles = POCRE SNPs. **(c)** Zoom-in of chromosome 7 (20.8 – 22.0 Mb) showing GWA peak in *aus*; gold dots represent SNP p-values from GWA analysis using the unimputed HDRA SNP data set; black dots represent SNPs from imputed SNP dataset; red dots indicate MS-SNPs using unimputed (SNP-7:21540960) and imputed SNP dataset (SNP-7:21564134); horizontal red bar indicates region of GWA peak (#87). **(d)** Haplotype analysis of 31 Kb region containing the MS-SNPs; blue boxes = reference (Nipponbare) alleles; yellow boxes = alternate alleles; red asterisks indicate MS-SNPs; boxes to right indicate number of lines carrying each haplotype within a subpopulation. **(e)** Quantile boxplot shows phenotypic distribution of Cd_shoot content for all haplotypes found in the INDICA clade which contains three *aus*-specific haplotypes.
